# Cyproterone acetate acts as a disruptor of the aryl hydrocarbon receptor

**DOI:** 10.1038/s41598-021-84769-7

**Published:** 2021-03-09

**Authors:** Chih-Shou Chen, Guan-Lun Gao, Dong-Ru Ho, Chih-Yi Lin, Yu-Ting Chou, Shan-Chun Chen, Min-Cong Huang, Wen-Ya Kao, Jyan-Gwo Joseph Su

**Affiliations:** 1 Division of Urology, Department of Surgery, Chang Gung Memorial Hospital, Chiayi, Taiwan, ROC; 2 Department of Biochemical Science and Technology, National Chiayi University, Chiayi, 60004 Taiwan, ROC; 3 Department of Biological Resources, National Chiayi University, Chiayi, 60004 Taiwan, ROC

**Keywords:** Cell signalling, Cell biology, Medical research, Urology

## Abstract

Prostate cancer is a major cause of death in males. Cyproterone acetate (CPA), the steroidal anti-androgen for part of androgen deprivation therapy, may block the androgen-receptor interaction and then reduce serum testosterone through its weak anti-gonadotropic action. In addition to CPA inducing hepatitis, CPA is known to cause liver tumors in rats also. Aryl hydrocarbon receptor (AhR) is a cytoplasmic receptor and regulates multiple physiological functions. CYP1A1 is an AhR-targeted gene. We found that CPA induced CYP1A1 expression, transcriptional activity of the aryl hydrocarbon response element (AHRE), and the nuclear localization of AhR in mouse Hepa-1c1c7 cells. However, CPA suppressed CYP1A1 mRNA expression and the transcriptional activity of AHRE in human HepG2 and MCF7 cells, and also decreased AhR ligand-induced CYP1A1 protein expression and transcriptional activity of AHRE in HepG2 cells. In summary, CPA is an AhR agonist in mouse cells, but an AhR antagonist in human cells. Accordingly, CPA potentially plays a role as an endocrine disruptor of the AhR. This study helps us to understand why CPA induces acute hepatitis, gene mutation, and many other side effects. In addition, it may trigger further studies investigating the relationships between CPA, glucocorticoid receptor and castration-resistant prostate cancer in the future.

## Introduction

Cyproterone acetate is used in hormone replacement therapy and several different types of cancer^1,2^. Combinations of antiandrogens and LHRH analogs are effective in lowering testosterone to castration level, but might also have significant impact on quality of life in patients with prostate cancer owing to osteoporosis, loss of sexuality, and muscle mass, and for this reason, total androgen block has been less adopted in the treatment of prostate cancer due to higher toxicity rate and decreased quality of life with complete androgen blockade^3^. Of course, second line antiandrogen- or androgen receptor-signaling inhibitors (ARSi) such as abiraterone, enzalutamide, apalutamide and darolutamide have an even more important role in the control of prostate cancer, which can be used earlier in hormonal-sensitive non-metastatic or metastatic chemo-naïve prostate cancer^4–10^. However, neoadjuvant hormonal therapy (NHT) for high-risk prostate cancer is still a challenge for urologists. The EMPaCT group study revealed that 403 NHT before radical prostatectomy could achieve a significantly decreased prostate Cancer-related death^11,12^, and although NHT could combine standard androgen deprivation therapy with ARSi or 2nd line antiandrogen^13,14^, the cost is expensive, so short-term NHT with less expensive antiandrogen might be a choice in achieving the same purpose. Antiandrogen is not expensive and is well-tolerated by patients with prostate cancer, being of two types, steroidal (cyproterone acetate) or nonsteroidal (bicalutamide, hydroxy-flutamide, and nilutamide)^15^. These antagonists prevent the activation of the androgen receptor (AR) and androgen-induced conformational changes. Survival after LHRH agonist treatment is equivalent to that after orchiectomy, but survival rate may be lower with use of a nonsteroidal antiandrogen^16^. Cyproterone acetate, the steroidal anti-androgen, can block androgen-receptor interaction and reduce serum testosterone through its weak anti-gonadotropic action. It has been referred to as the only anti-hormone that causes complete androgen blockade as monotherapy^17^; however, due to potential adverse effects of CPA including hepatitis and liver tumor, CPA needs to be understood in more detail.

The aryl hydrocarbon receptor (AhR) was first found to be activated by dioxin, and is involved in detoxification for the xenobiotics. When xenobiotics, such as polycyclic aromatic hydrocarbons (PAHs) enter the body, they bind and then activate aryl hydrocarbon receptor (AhR) in cells. The ligand-bound AhR translocates into the nucleus and binds to aryl hydrocarbon receptor nuclear translocator protein (ARNT) to form an active nuclear transcription factor, binding to aryl hydrocarbon receptor response element (AHRE), resulting in the transcription of AhR-sensitive genes^18,19^. ARNT is an essential partner of AhR in the active form, with AhR inducing drug-metabolic enzymes in all of the three stages (*phase I, II, and III*) of the detoxification process^20^. The functions of phase I, II, and III include the introduction of a hydroxyl group on the aryl hydrocarbons, the conjugation with glutathione, sulfate, or glucuronide, and the elimination of phase metabolites from cells respectively. Both groups of enzymes, cytochrome p450 (CYP) and aldoketo reductases (AKRs) belong to phase I drug-metabolizing enzymes^21^; however, some reactive intermediaries of phase I might interact with DNA and other cellular components, resulting in toxic effects. Accordingly, CYP 1A1, one of the major phase I enzymes, is regarded as a carcinogen-metabolizing enzyme. CYP1A1 is the best-known AhR-sensitive target; therefore, the expression level of CYP1A1 is often used as an indicator for activation of the AhR.

Although the role of the AhR in endocrinology has not yet been clarified, an endogenous ligand of AhR, 2-(1H-Indol-3-ylcarbonyl)-4-thiazolecarboxylic acid methyl ester (ITE), has been isolated from lung tissue^22^ and confirmed to reduce colitis through induction of regulatory T cells and treat autoimmune diseases^23^, also suppressing angiogenic responses of human umbilical artery endothelial cells in vitro through an AhR-dependent pathway^24^.

Our data indicates that cyproterone acetate activated AhR and induced the expression of CYP1A1 in mouse cells, but antagonized the AhR and decreased the transcription of CYP1A1 expression in human cells. The effects of cyproterone acetate on the CYP1A1 expressions were mediated by the AhR signal. In this article we show that cyproterone acetate is an AhR agonist in mouse cells, but an AhR antagonist in human cells.

## Results

### Cyproterone acetate caused minor decreases of cell vitality.

**Figure 1:**
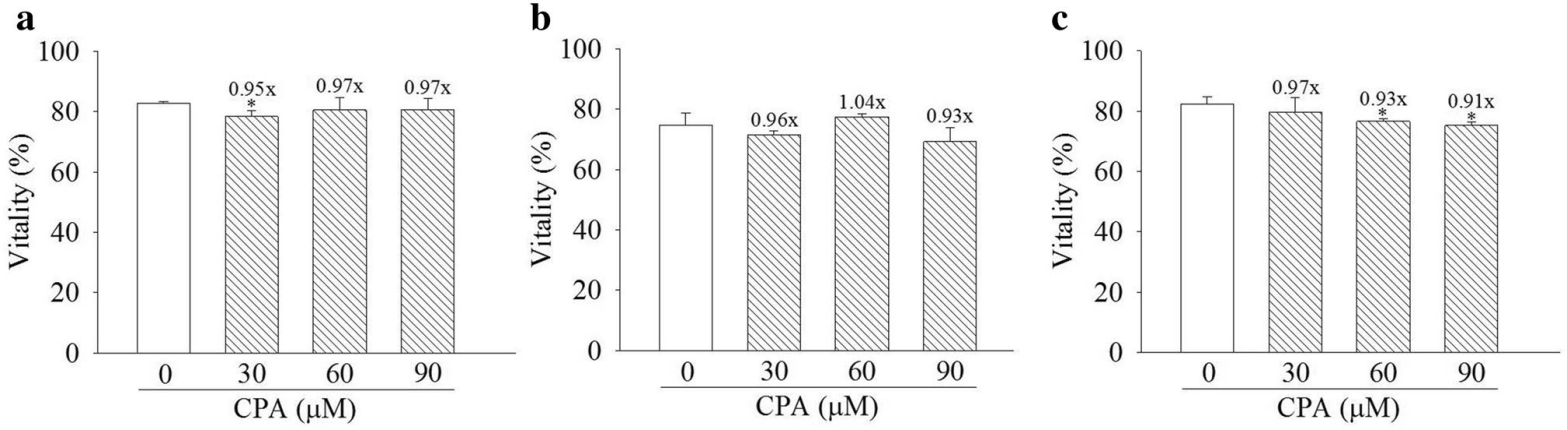
Vitality assay of cyproterone acetate (CPA)-treated cells. (**a**) HepG2, (**b**) MCF7 and (**c**) Hepa-1c1c7 cells were treated with CPA (30, 60 and 90 μM) for 48 h. Vitality rates are indicated by the percentage of healthy cells. Multiples of healthy cells among treated cells relative to those in the control are indicated at the top of bar. * Indicates comparison with DMSO-treated cells of healthy cells. Results are expressed as the mean ± SD, *n* = 3. *p < 0.05.

HepG2, MCF7, and Hepa-1c1c7 cells were treated with cyproterone acetate (30, 60 and 90 μM, equivalent to 12.51, 25.02 and 37.53 μg/ml respectively) for 48 h. Under treatment with cyproterone acetate for the same condition did not cause significant decrease of cell viability of both HepG2 and MCF7 cells (Fig. 1a,b). Treatment with 90 μM cyproterone acetate for 48 h caused only minor decrease, 9%, of cell viability of Hepa-1c1c7 cells (Fig. 1c). In human prostate cancer, the usual dosage of cyproterone acetate prescribed to patients is 50 mg thrice daily (range allowable between 50–300 mg per day).

### Cyproterone acetate stimulates expressions of the CYP1A1 mRNA and protein in mouse cells

**Figure 2:**
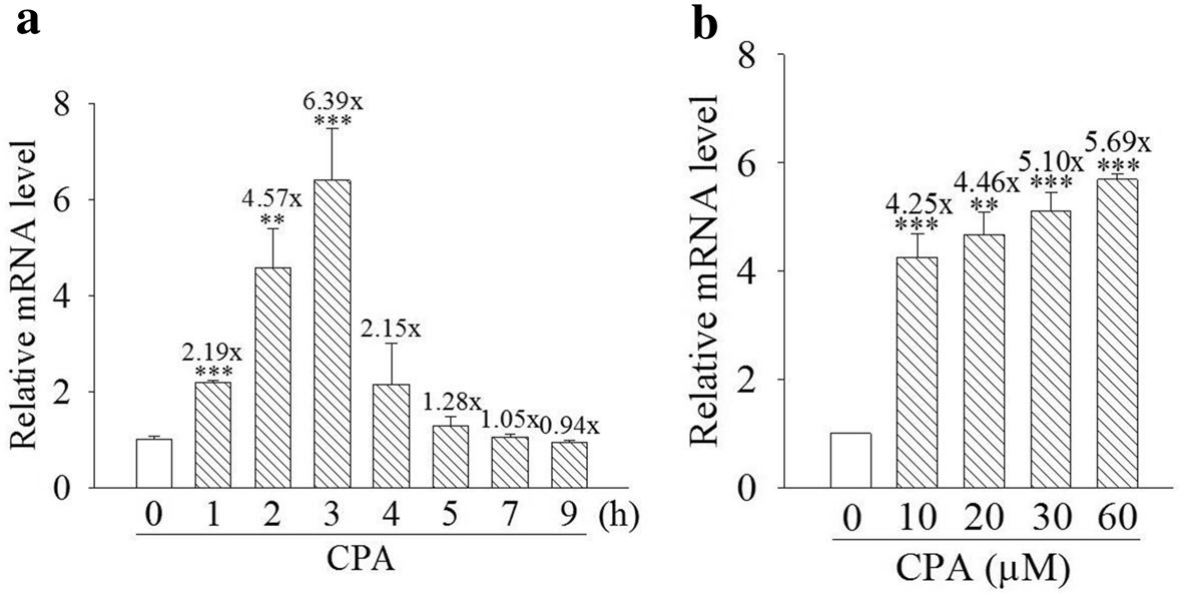
Expression profiles of cytochrome P450 1A1 (CYP1A1) mRNA induced by cyproterone acetate (CPA) in mouse cells. (**a**) Hepa-1c1c7 cells were treated with CPA (30 μM) for 1–9 h. (**b**) Hepa-1c1c7 cells were treated with CPA (10, 20, 30, and 60 μM) for 3 h. Total RNA was harvested for the analysis. The expression of CYP1A1 mRNA was analyzed by quantitative PCR, as described in “Materials and methods”. Results are expressed as the mean ± SD, *n* = 3. **p < 0.01, and ***p < 0.001.

The induction of CYP1A1 mRNA expression was detectable after 1 h of treatment with cyproterone acetate (30 μM) (Fig. 2a). Treatment with cyproterone acetate reached a maximum level at 3 h up to 6.39-fold induction of mRNA expression, and distinctly decreased thereafter. In the dosage study, treatments with 60 μM cyproterone acetate for 3 h still did not reach the maximal induction of CYP1A1 mRNA expression (Fig. 2b).

**Figure 3:**
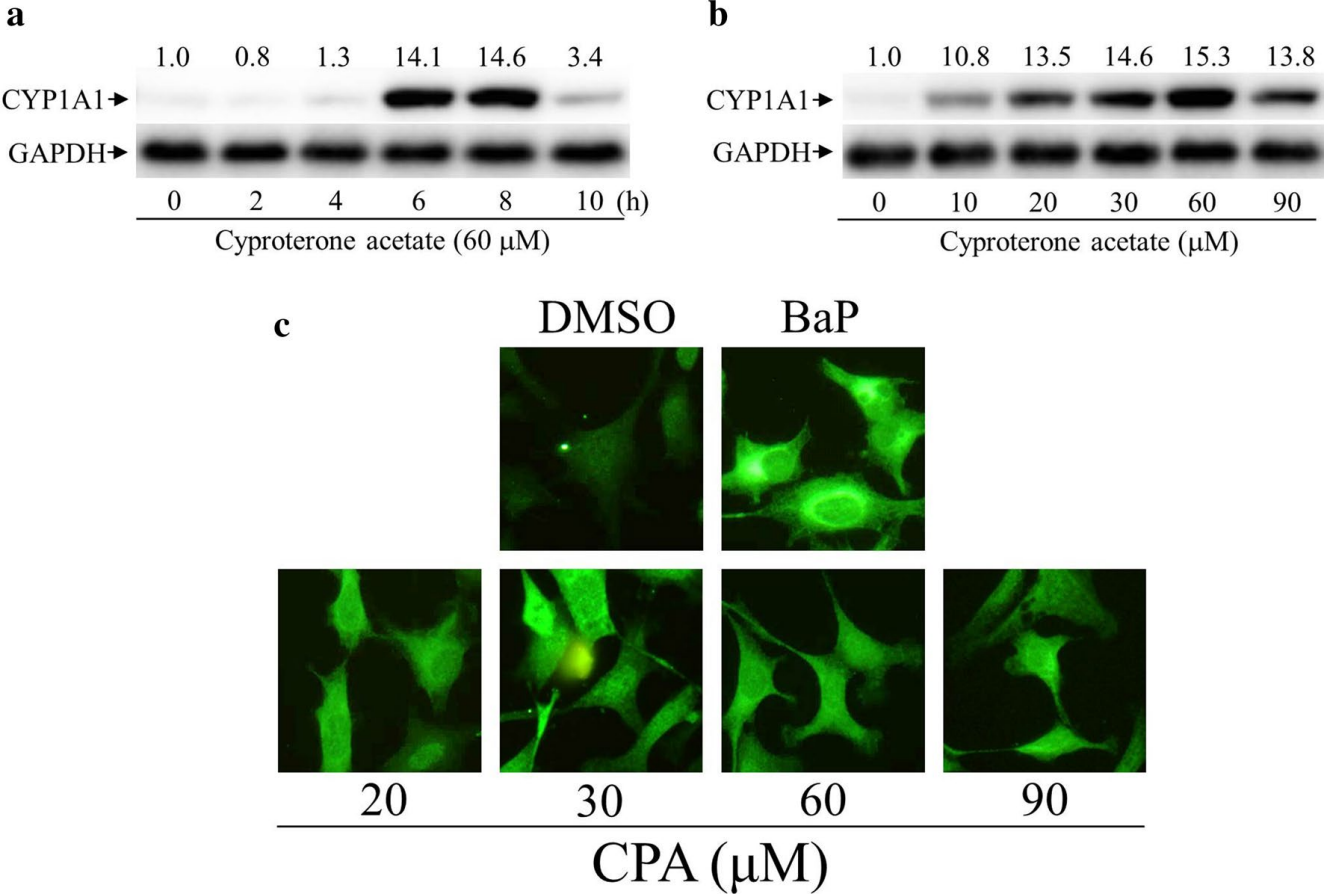
Expression profiles of cytochrome P450 1A1 (CYP1A1) protein induced by cyproterone acetate (CPA) in mouse cells. (**a**) Hepa-1c1c7 cells were treated with CPA (60 μM) for 2–10 h. (**b**) Hepa-1c1c7 cells were treated with CPA (10–90 μM) for 6 h. Expression of the CYP1A1 protein was analyzed by Western blots. The CYP1A1 protein levels revealed by Western blotting were quantified and standardized against the amounts of β-actin or GAPDH protein. The relative levels of CYP1A1 expression are indicated on the top of the bands. (**c**) CPA and benzo[*a*]pyrene (BaP)-induced CYP1A1 expression as revealed by immunofluorescence images. Hepa-1c1c7 cells were treated with CPA (20–90 μM) and BaP (10 μM) for 6 h. Afterward, cells were fixed with ethanol. Expression of the CYP1A1 protein was probed using an antibody against CYP1A1, as revealed by fluorescence of a rabbit polyclonal secondary antibody to rabbit IgG-H&L (DyLight 488). Fluorescence emitted by cells was viewed using a fluorescence microscope, equipped with optical filters at excitation/emission wavelengths of 493/518 nm.

The induction of CYP1A1 protein expression was detectable after 4 h treatment with cyproterone acetate (60 μM), reaching a maximum level up to 14.6-fold at 8 h treatment, and distinctly decreased thereafter (Fig. 3a). In the dosage study, treatments with cyproterone acetate (60 μM) for 6 h reached a maximal induction of CYP1A1 protein expression up to 15.3-fold (Fig. 3b). The expression of CYP1A1 was further examined by immuno-cellular fluorescence staining. Benzo[a]pyrene (BaP) is a polycyclic aromatic hydrocarbon (PAH), and a potent AhR ligand^25^. Hepa-1c1c7 cells were treated with cyproterone acetate (20–90 μM) and BaP (10 μM) for 6 h, and its CYP1A1 expression was revealed by immunofluorescence images (Fig. 3c). BaP-induced CYP1A1 expression was used as a positive control.

### Cyproterone acetate stimulates transactivation activity of the AhR in mouse cells

To identify cyproterone acetate-induced transactivation activity of the AhR, the pAhRDtkLuc3 reporter plasmid containing three AHRE motifs and the RSV-*lac*Z reporter plasmid were transfected into the Hepa-1c1c7 cells.

**Figure 4:**
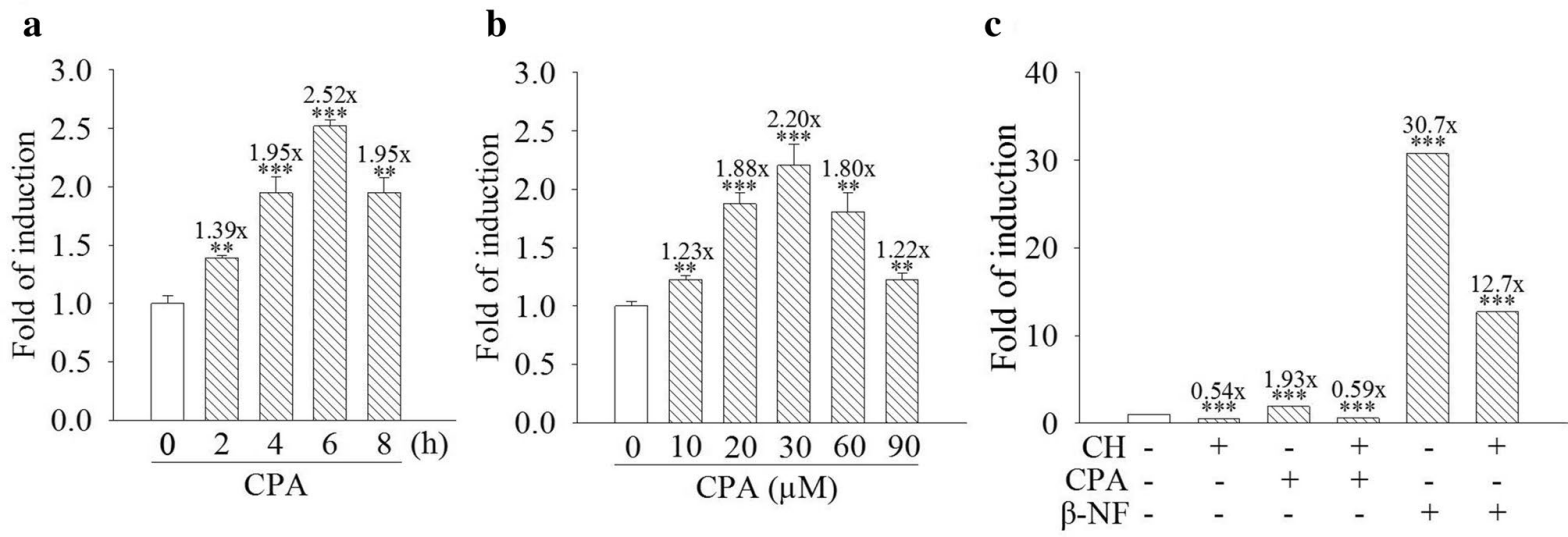
Effect of cyproterone acetate (CPA) on the transactivation activity of the aryl hydrocarbon response element (AHRE) in mouse cells. Reporter plasmids, pAhRDtkLuc3 and RSV-*lac*Z, were transfected into Hepa-1c1c7 cells. (**a**) Hepa-1c1c7 cells were treated with CPA (30 μM) for 2–8 h. (**b**) Hepa-1c1c7 cells were treated with CPA (10, 20, 30, 60, and 90 μM) for 6 h. (**c**) Hepa-1c1c7 cells were pretreated with 10 μM CH-223191 (CH) for 1 h, followed by treatment with 30 μM CPA and 1 μM β-NF for 6 h. At the end of incubation with the test compounds, cells were harvested, and cell lysates were collected for an activity assay of luciferase and β-galactosidase. Results are expressed as the mean ± SD, *n* = 3. **p < 0.01, and ***p < 0.001.

Hepa-1c1c7 cells with the reporter plasmids were treated with 30 μM cyproterone acetate for 2–8 h. Cyproterone acetate increased the transcriptional activity of the AHRE by up to 2.5-fold at 6 h treatment (Fig. 4a). Treatment with cyproterone acetate (10–90 μM) for 6 h dose-dependently induced the transcriptional activity of the AHRE and reached a maximal induction, 2.2-fold, by 30 μM treatment (Fig. 4b).

### Cyproterone acetate’s induction of CYP1A1 expression is AhR-dependent

**Figure 5:**
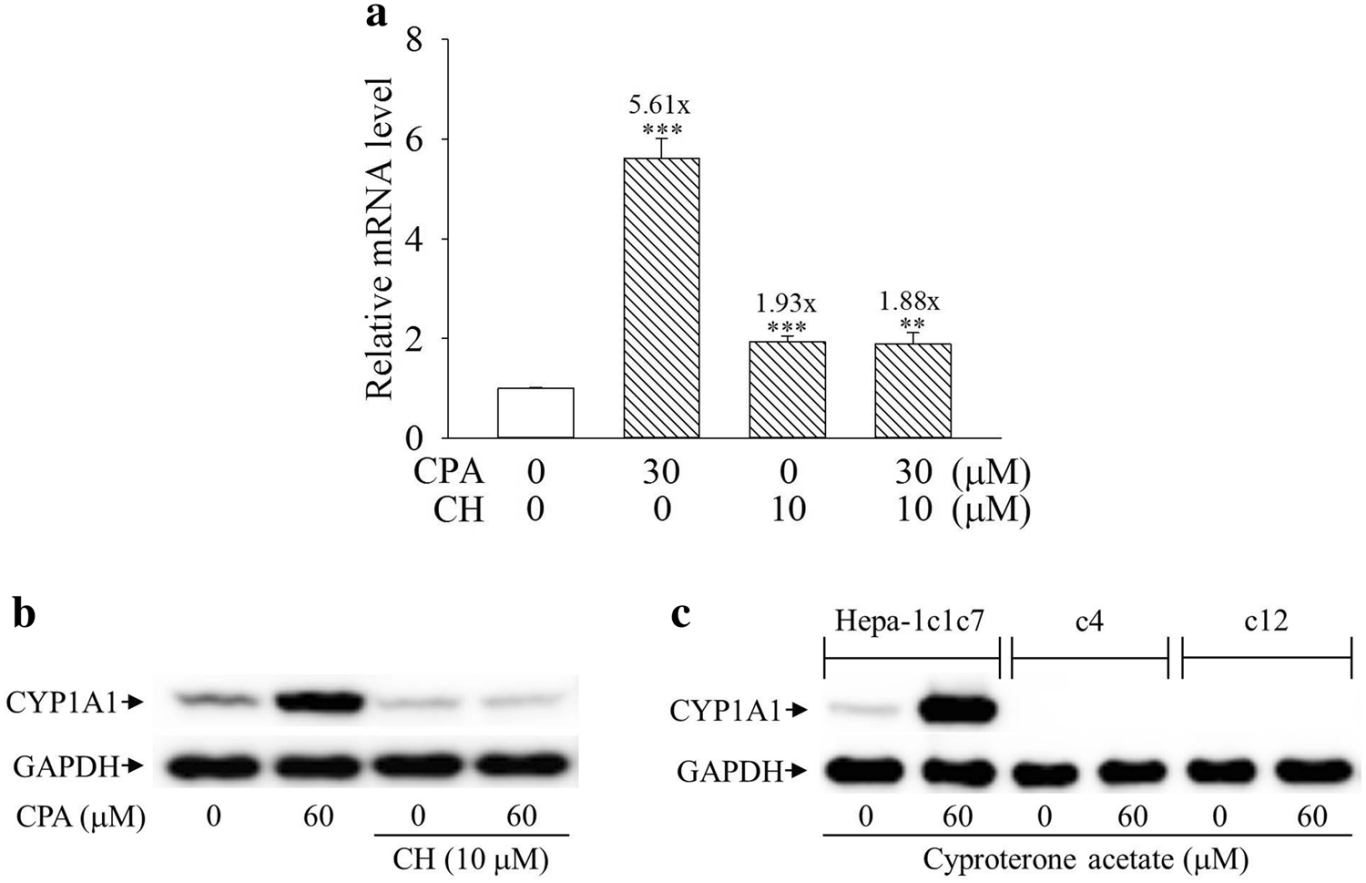
Effect of the aryl hydrocarbon receptor (AhR) signal on the expression of cytochrome P450 1A1 (CYP1A1) induced by cyproterone acetate (CPA) in mouse cells. (**a**) Hepa-1c1c7 cells were pretreated with 10 μM CH-223191 (CH) for 1 h, followed by treatment with 30 μM CPA for 3 h. The expression of CYP1A1 mRNA was analyzed by quantitative PCR, as described in “Materials and methods”. Results are expressed as the mean ± SD, *n* = 3. ** *p* < 0.01, and *** *p* < 0.001. (**b**) Hepa-1c1c7 cells were pretreated with 10 μM CH-223191 (CH) for 1 h, followed by treatment with CPA for 6 h. The CYP1A1 protein expression of their cell lysates was analyzed by Western blots. (**c**) Hepa-1c1c7, c4 (B13NBii1), and c12 (B15ECiii2) cells were treated with CPA (60 μM) for 6 h. The CYP1A1 protein expression of their cell lysates was analyzed by Western blots.

Figure 4a,b show that cyproterone acetate induced the transcriptional activity of AHRE. In order to further examine whether the AhR signal could mediate cyproterone acetate’s induction of CYP1A1 expression, AhR antagonist was applied to block the cyproterone acetate-induced of CYP1A1 expression. CH-223191 is a synthetic AhR antagonist^26^ and β-NF is a synthetic AhR agonist, used as a positive control for the induction of AhR activity. Both cyproterone acetate and β-NF increased the transcriptional activity of the AHRE, but their inductions were blocked by CH-223191 (Fig. 4c). When cells were treated with cyproterone acetate together with CH-223191, both cyproterone acetate-induced CYP1A1 mRNA and protein expressions were highly suppressed in Hepa-1c1c7 cells (Fig. 5a,b). In addition, AhR signal-deficient Hepa-1c1c7-derivative cells, c4 and c12, were applied. CYP1A1 expression was highly induced by cyproterone acetate in Hepa-1c1c7 cells, but not in c4 and c12 cells (Fig. 5c).

**Figure 6:**
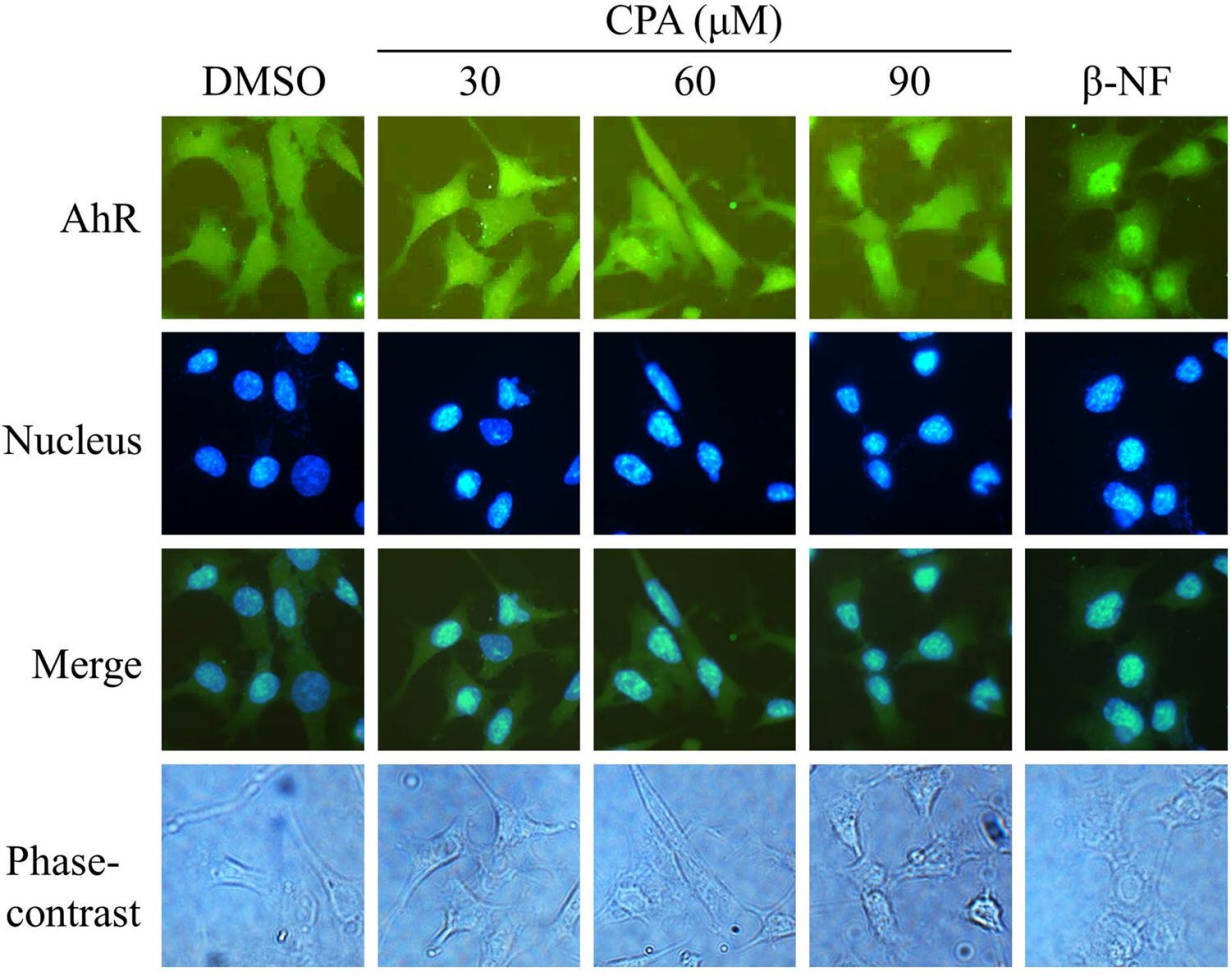
Induction of nuclear localization of the aryl hydrocarbon receptor (AhR) by cyproterone acetate (CPA) in mouse cells. Hepa-1c1c7 cells were treated with CPA (30, 60 and 90 μM) and β-NF (10 μM) for 2 h, and then cells were fixed with 4% formaldehyde, and nuclei were stained with Hoechst 33342 (5 μg/ml). Expression of the AhR protein was probed using an antibody against the AhR, as revealed by the fluorescence of a rabbit polyclonal secondary antibody to goat IgG-H&L (DyLight 488). Fluorescence emitted by DyLight 488 was viewed using a fluorescence microscope, equipped with optical filters at excitation/emission wavelengths of 493/518 nm. Fluorescence emitted by Hoechst 33342 was viewed using a fluorescence microscope, equipped with optical filters at excitation/emission wavelengths of 346/460 nm. Fluorescence images for the AhR and nucleus were merged.

To analyze whether the AhR was activated by cyproterone acetate, nuclear localization of the AhR was monitored by an immunofluorescence image. The AhR translocated to the nucleus when Hepa-1c1c7 cells were treated with cyproterone acetate (30, 60, and 90 μM) and β-NF (10 μM) for 2 h (Fig. 6). β-NF is a synthetic AhR agonist^22^ and the β-NF-induced nuclear localization of AhR was used as a positive control. The location of the nucleus was revealed by the fluorescence dye, Hoechst 33,342.

### Cyproterone acetate suppresses CYP1A1 mRNA expression and the AhR ligand-induced CYP1A1 protein expression in human cells

**Figure 7:**
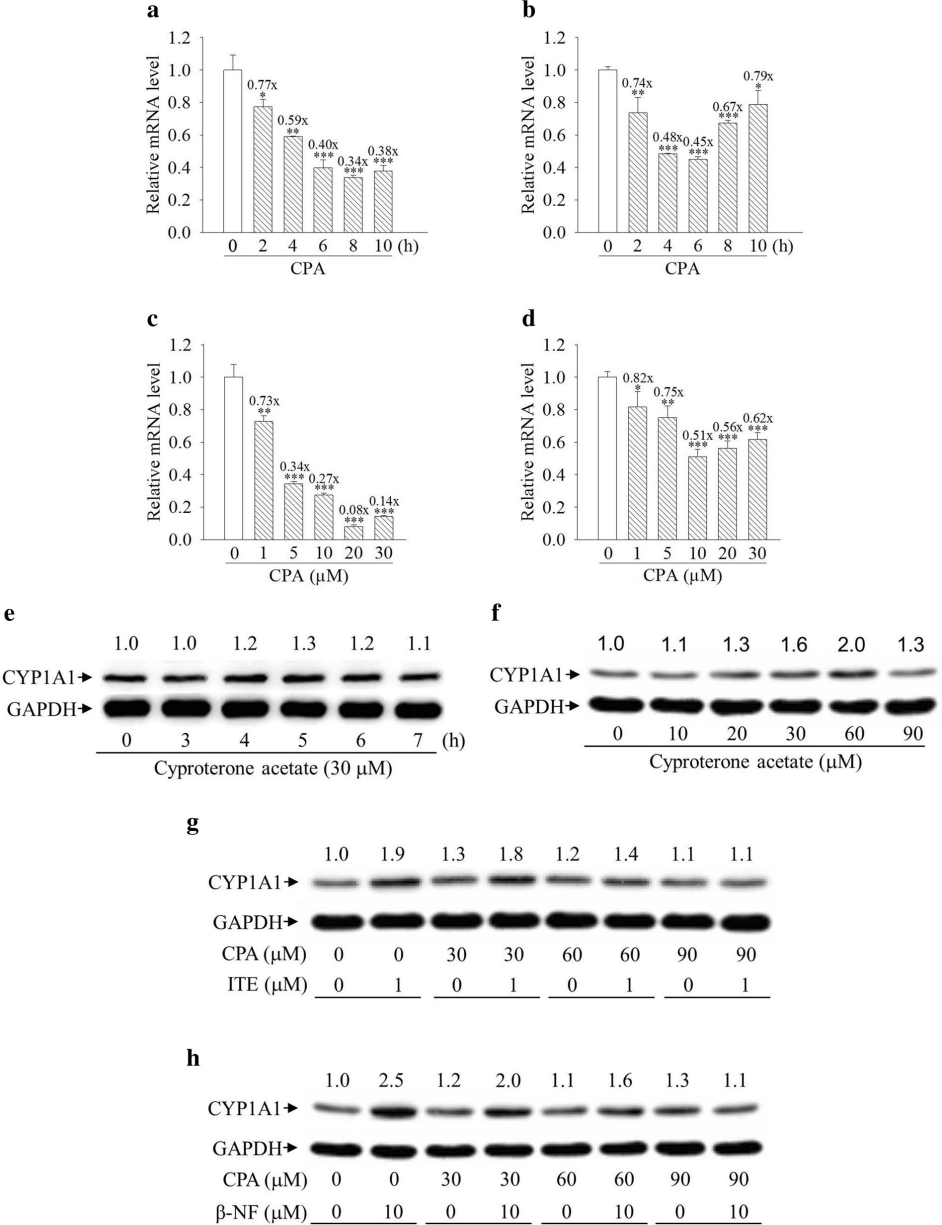
Expression profiles of cytochrome P450 1A1 (CYP1A1) mRNA and protein induced by cyproterone acetate (CPA) in human cells. (**a**,**b**) HepG2 and MCF7 cells were treated with CPA (10 μM) for 2–10 h. (**c**,**d**) HepG2 and MCF7 cells were treated with CPA (1–30 μM) for 6 h. Total RNA was harvested for the analysis. The expression of CYP1A1 mRNA was analyzed by quantitative PCR, as described in “Materials and methods”. Results are expressed as the mean ± SD, *n* = 3. *p < 0.05, **p < 0.01, and ***p < 0.001. (**e**) HepG2 cells were treated with CPA (30 μM) for 3–7 h. (**f**) HepG2 cells were treated with CPA (10–90 μM) for 5 h. (**g**,**h**) HepG2 cells were pretreated with 30–90 μM CPA for 1 h, followed by treatment with 1 μM ITE for 5 h and 10 μM β-NF for 6 h. Expression of the CYP1A1 protein was analyzed by Western blots. The CYP1A1 protein levels revealed by Western blotting were quantified and standardized against the amount of GAPDH protein. The relative levels of CYP1A1 are indicated on the top of the bands.

Treatment with cyproterone acetate suppressed CYP1A1 mRNA expression with time course- and dose-dependency in HepG2 and MCF7 cells. The maximal suppression of CYP1A1 mRNA expression was detected at 8 and 6 h of treatment with cyproterone acetate (10 μM) in HepG2 and MCF7 cells respectively, and thereafter, the extent of suppression effect was decreased (Fig. 7a,b). Treatment of cyproterone acetate (20 and 10 μM) for 6 h in HepG2 and MCF7 cells respectively caused the maximal suppression of CYP1A1 mRNA expression (Fig. 7c,d), and treatment with higher doses of cyproterone acetate did not cause further suppression.

Treatment with 30 μM cyproterone acetate for 3–7 h did not distinctly increase CYP1A1 protein expression in HepG2 cells (Fig. 7e). Treatment with 10–90 μM cyproterone acetate for 5 h also did not distinctly increase CYP1A1 protein expression in HepG2 cells (Fig. 7f).

ITE is an endogenous AhR ligand, and β-naphthoflavone (β-NF) is a synthetic AhR ligand. Co-treatment of 30–60 μM cyproterone acetate with either 1 μM ITE or 10 μM β-NF reduced both ITE- and β-NF-induced CYP1A1 protein expression (Fig. 7g,h).

### Cyproterone acetate suppresses transactivation activity of the AhR in human cells

**Figure 8:**
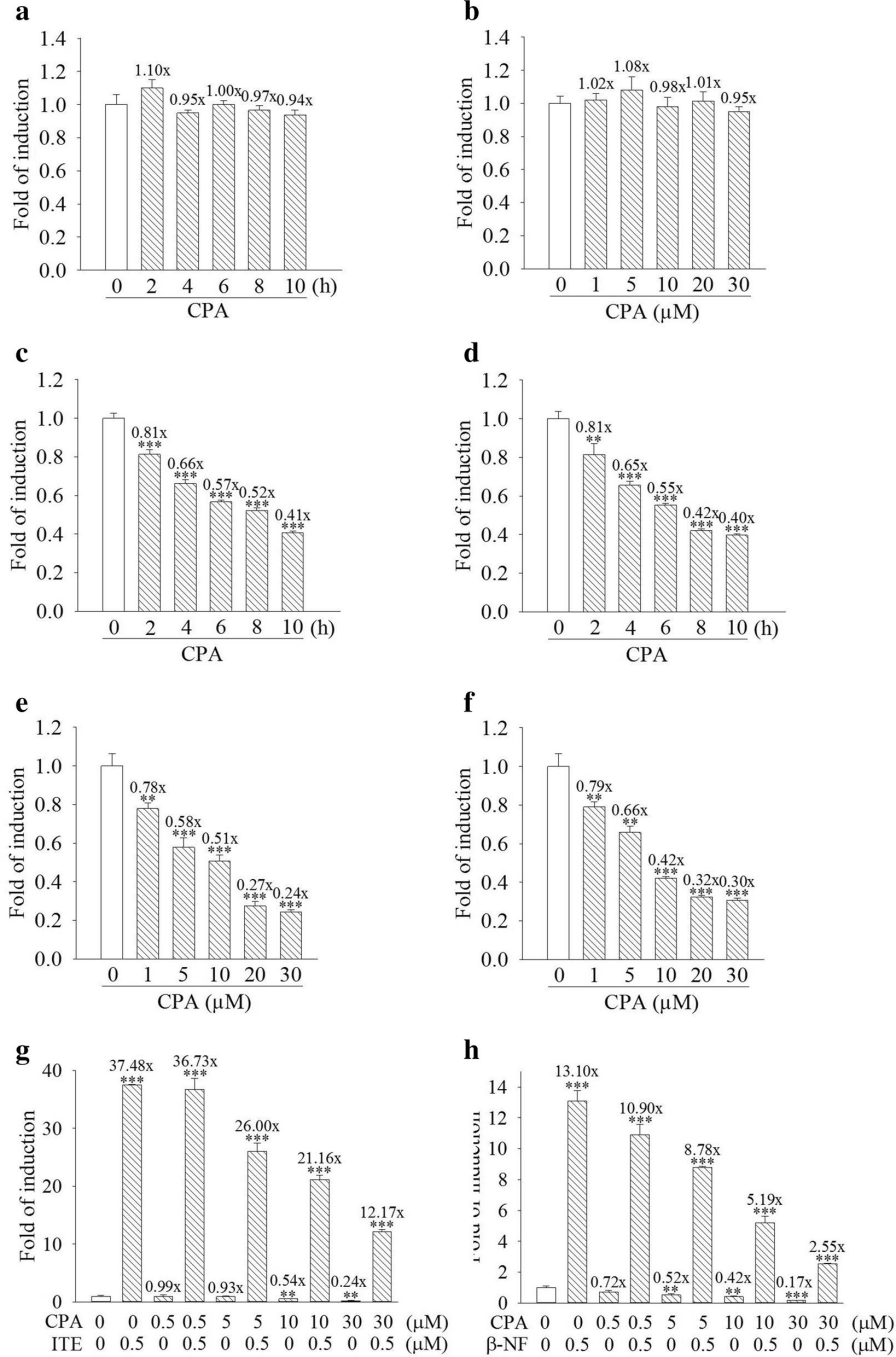
Effect of cyproterone acetate (CPA) on the transactivation activity of the aryl hydrocarbon response element (AHRE) in human cells. Reporter plasmids, (**a**,**b**) pTAL-Luc and RSV-*lac*Z or (**c-h**) pAhRDtkLuc3 and RSV-*lac*Z, were transient transfected into HepG2 and MCF7 cells for 18 h, and then cells were treated with the test compounds. (**a**) HepG2 cells were treated with CPA (10 μM) for 2–10 h. (**b**) HepG2 cells were treated with CPA (1–30 μM) for 6 h. (**c**,**d**) HepG2 and MCF7 cells were treated with CPA (10 μM) for 2–10 h. (**e**,**f**) HepG2 and MCF7 cells were treated with CPA (1–30 μM) for 6 h. (**g**,**h**) HepG2 cells were pretreated 0.5–30 μM CPA for 1 h, followed by treatment with 0.5 μM ITE for 6 h and 0.5 μM β-NF for 9 h respectively. At the end of incubation with the test compounds, cells were harvested and cell lysates were collected for an activity assay of luciferase and β-galactosidase. Results are expressed as the mean ± SD, *n* = 3. **p < 0.01, and ***p < 0.001.

Plasmid of pTAL-Luc is a control reporter driven by a minimal tk promoter without AHRE, and will not respond to the AhR ligand. Treatment with cyproterone acetate (10 μM, 2–10 h) or (1–30 μM, 6 h), did not significantly regulate tk promoter activity of pTAL-Luc in the HepG2 cells transfected with the reporter plasmids of pTAL-Luc and RSV-*lac*Z (Fig. 8a,b). Treatment with cyproterone acetate (10 μM) for a 2–10 h time course dependently decreased the transcriptional activity of the AHRE in both HepG2 and MCF7 cells transfected with the reporter plasmids of pAhRDtkLuc3 and RSV-*lac*Z (Fig. 8c,d). Treatment with cyproterone acetate (1–30 μM) for 6 h dose-dependently decreased the transcriptional activity of the AHRE in both HepG2 and MCF7 cells transfected with the reporter plasmids of pAhRDtkLuc3 and RSV-*lac*Z (Fig. 8e,f). When HepG2 cells carrying the reporter plasmids of pAhRDtkLuc3 and RSV-*lac*Z were co-treated with 0.5–30 μM cyproterone acetate plus either 0.5 μM ITE or 0.5 μM β-NF, cyproterone acetate dose-dependently reduced both ITE- and β-NF-induced transcriptional activity of the AHRE (Fig. 8g,h).

## Discussion

Cyproterone acetate has been shown to induce clinically acute hepatitis^27^ and gene mutation in rats^28^. There is a variety of hepatotoxic reactions reported with cyproterone acetate^29^, and severe drug-induced hepatotoxicity might develop during treatment with cyproterone acetate. There is also suspected cross-hepatotoxicity between cyproterone acetate and other antiandrogens^30^; besides, cyproterone acetate is known to cause liver tumors in rats. The drug has been identified recently as a mutagen in the liver of female transgenic lambda Laci (Big Blue) rats at high doses after an expression time of 6 weeks^31^. It is also noted to be mitogenic, tumorigenic and induces DNA-adducts and DNA-repair synthesis in rat liver^28^. To make better use of this effective antiandrogen, it is necessary to understand it better.

There are three stages of AhR-induced detoxification process (*phases I, II, and III*), and AhR induces drug-metabolic enzymes in every stage^20^. CYP1A1 is one of the major phase I enzymes, and the most well-known of the AhR-targeted genes. It introduces oxygen and hydroxyl groups on the aryl hydrocarbon, resulting in increasing solubility of the aryl hydrocarbon. However, the metabolic intermediate forms might be active and interact with cellular components including DNA and resulting in mutagenesis^32^. For example, benzo[a]pyrene (BaP) is metabolized by CYP1A1 into a reactive intermediate B[a]P diol epoxide (BPDE)^32,33^ regarded as a carcinogenic derivative, due to its binding to DNA. Accordingly, CYP1A1 is regarded as a carcinogen-metabolizing enzyme.

We found that cyproterone acetate time course- and dose-dependently induced CYP1A1 mRNA and protein expression in mouse Hepa-1c1c7 cells. CYP1A1 mRNA expression was induced one hour after treatment of cyproterone acetate. The induced CYP1A1 protein expression reached maximal level after 6 h treatment of cyproterone acetate.

AhR is a cytoplasmic receptor localized in the cytoplasma, and upon activation, it translocates into the nucleus and plays a role as a transcription factor, and in turn, transcribes its target genes, including CYP1A1. AHRE is the target genome sequence of the activated AhR. The AhRDtkLuc3 reporter plasmid containing three AHRE motifs was used to indicate whether AhR is activated. Data indicated that cyproterone acetate time course- and dose-dependently stimulates transactivation activity of the AhR in mouse Hepa-1c1c7 cells.

The antagonist of AhR, CH-223191, was applied to examine whether cyproterone acetate binds directly to AhR. CH-223191 suppressed the cyproterone acetate’s induction of AHRE-mediated transcriptional activity, CYP1A1 mRNA and protein expressions. Both the c4 and c12 cell lines are Hepa-1c1c7 cell derivatives with deficiencies in ARNT and reduced AhR protein level respectively. Cyproterone acetate induced CYP1A1 protein expression in Hepa-1c1c7 cells, but did not induce it in c4 and c12 cells. In addition, cyproterone acetate was able to induce the translocalization of AhR from cytosol into nucleus. All of these results further confirmed that the AhR mediated the cyproterone acetate action in inducing CYP1A1 expression in mouse Hepa-1c1c7 cells. However, when the cyproterone acetate was applied in human HepG2 and MCF7 cells, it dose- and time course-dependently decreased the CYP1A1 mRNA expression. ITE and β-NF are endogenous and synthetic AhR ligands respectively. Although cyproterone acetate did not decrease CYP1A1 protein expression levels, it suppressed ITE- and β-NF-induced CYP1A1 protein expression. In addition, cyproterone acetate dose- and time course-dependently decreased AHRE transactivation activity in both human HepG2 and MCF7 cells. These results indicate that cyproterone acetate antagonizes the AhR activity in human cells. Similar results were obtained in HepG2 and MCF7, indicating that it is not cell-specific. The differential function of cyproterone acetate on the AhR activity between human and mouse cells is likely due to the species-specificity. The opposite effect of CPA on AhR activity in human cells compared to murine cells provides interesting information for further studies in the protein sequence and structure of AhR related to the ligand-activation or -inhibition.

We have demonstrated the crosstalk between the AhR and glucocorticoid receptor (GR)^34^. Dexamethasone is a synthetic agonist of glucocorticoids. AhR agonist enhances dexamethasone-induced transactivation activity of the GR^34^. This indicates that the cyproterone acetate-disrupted AhR also has potential to interact with the dexamethasone-activated GR. Accordingly, cyproterone acetate also potentially interferes with GR-targeted gene expressions. This finding also highlights the potential role of CPA in management of increasing expression of glucocorticoid receptor, which might be the reason that castration-resistant prostate cancer is refractory to the treatment of enzalutamide^35,36^. Targeting the glucocorticoid receptor with additional antiandrogen might further mitigate castration-resistance in prostate cancer therapy.

ITE is a potent endogenous ligand of AhR, first isolated from lung tissue^22^, and is involved in several physiological roles including suppressing autoimmune diseases^23^ and angiogenic responses of human umbilical artery endothelial (HUAECs) cells in vitro^24^. Triple-negative breast cancer (TNBC) is the most aggressive breast cancer subtype. JAG1-NOTCH1 signaling involves the aggressiveness of TNBC. Piwarski and his colleagues demonstrated that ITE reduces the expression of JAG1 in the amount of Notch 1 intracellular domain (NICD1) in TNBC MDA-MB-231 cells^37^, and theoretically, will negatively regulate the development of the TNBC. Our results indicate that cyproterone acetate suppressed ITE-induced AhR activity in human cells; thus, cyproterone acetate not only interferes with the AhR-regulated physiological functions but also reverses the functions of the endogenous ligand, ITE, including the ITE-suppression of TNBC.

Recently it has been shown that AhR plays multiple physiological roles, including the control of cytokine and chemokines, reproductive steroidogenesis mediated by the AhR-target gene CYP19, and the regulation of vascular physiological functions^38,39^. AhR also cross-talks with antioxidant regulator Nrf2^40^.

In summary, we demonstrated that cyproterone acetate is an AhR agonist in mouse cells, but an AhR antagonist in human cells. Cyproterone acetate reversed the actions of the endogenous AhR ligand, ITE, thus potentially playing a role as an endocrine disruptor of the AhR. This study helps us understand why CPA induces acute hepatitis, gene mutation, and many other side effects. In addition, it should trigger further studies involving CPA, glucocorticoid receptor and castration-resistant prostate cancer.

## Materials

### Reagents and antibodies

Cyproterone acetate was obtained from Selleckchem (Houston, TX). CH-223191 and ITE was obtained from Tocris Bioscience (Minneapolis MN). β-NF and Hoechst 33342 was obtained from Sigma (St. Louis, MO). BaP was obtained from ChemService (West Chester, PA). Minimum essential medium (MEM) and MEMα and fetal bovine serum (FBS) were obtained from Gibco (Grand Island, NY). Antibody (Ab) against β-actin, GAPDH, AhR and human and mouse CYP1A1 (custom made) were obtained from Genetex Inc. (Irvine, CA). Ab against AhR was also obtained from Santa Cruz Biotechnology (Santa Cruz, CA). Donkey anti-goat immunoglobulin G (IgG)-horseradish peroxidase (HRP), goat anti-mouse IgG-HRP, and goat anti-rabbit IgG-HRP were obtained from Santa Cruz Biotechnology (Santa Cruz, CA). A rabbit polyclonal secondary Ab to goat IgG-H&L (DyLight 488) was obtained from Abcam (Cambridge, UK). Liposome was obtained from T-Pro Biotechnology (Ji-Feng Biotechnology, Taiwan R.O.C.).

### Cell culture

The mouse hepatoma cell lines, Hepa-1c1c7, c4 (B13NBii1), and c12 (B15ECiii2), the human breast adenocarcinoma MCF7 cells and the human hepatocellular carcinoma HepG2 cells were obtained from the American Type Tissue Collection (Rockville, MD). Both the c4 and c12 cell lines were derived from Hepa-1c1c7 and respectively had deficiency in Arnt and with reduced levels of the AhR protein. Hepa-1c1c7 cells and their derivatives along with MCF7 cells were maintained in MEMα medium. HepG2 cells were cultured in MEM medium. All of the culture medium were supplemented with 10% FBS, 2 mM L-glutamine, 100 units/ml penicillin, and 100 μg/ml streptomycin, then maintained at 37 °C in a 5% CO_2_/95% air environment. β-NF for the vitality assay was dissolved in methanol/ethyl acetate (2:3); otherwise, the test compounds were dissolved in dimethyl sulfoxide (DMSO). Agents were dissolved in dimethyl sulfoxide (DMSO).

### Vitality assay (analysis of the level of cellular thiols)

Cell vitality is evaluated by the changes in the intracellular levels of reduced thiols, as described previously^41^. Cells were stained with solution 5, including the fluorescence dyes of VitaBright-48, acridine orange, and propidium iodide (PI) **(**ChemoMetec A/S, Denmark), and analyzed using the fluorescence to determine various cell populations: the PI-negative cells (the healthy and unhealthy cells), and the PI-positive cells (the necrotic cells). VitaBright-48 stains viable cells in a fluorescence intensity-dependent manner for the level of thiols.

HepG2 and MCF7 cells were seeded at 4 × 10^5^ cells/6-cm dish overnight, followed by treatment with cyproterone acetate for a period of time. Hepa-1c1c7 cells were seeded at 2 × 10^5^ cells/6-cm dish overnight, followed by treatment with cyproterone acetate for a period of time. Afterwards, cells were suspended in trypsin, spun down with a centrifuge, and resuspended in 1 ml PBS. Solution 5 (1 μl) plus the cell suspension (19 μl) samples were mixed and loaded in NC-Slide A8 **(**ChemoMetec A/S, Denmark), and then fluorescence at the single cell level in the slide was analyzed and quantified with the NucleoCounter NC-3000 **(**ChemoMetec A/S, Denmark).

### Reverse-transcription polymerase chain reaction (RT-PCR) and quantitative PCR

HepG2, MCF7 and Hepa-1c1c7 cells were cultured at 8 × 10^5^, 8 × 10^5^ and 6 × 10^5^ cells respectively per 6-cm dish overnight, and then were treated with the test compounds. Total RNA of cells treated with the test compound was extracted using the NucleoZOL (MACHEREY–NAGEL GmbH & Co. KG, German). The complementary (c)DNA was reverse-transcribed from RNA using Magic RT cDNA synthesis kit (Bio-Genesis Technologies Inc., Taiwan) with oligo-dT (18) and random hexamer. The cDNA was amplified in the quantitative PCR with specific oligonucleotide primers for human CYP1A1 (GenBank: NM_000499), human GAPDH (GenBank: NM002046.5), mouse Cyp1a1 (GenBank: NM_009992), and mouse β-actin (GenBank: NM_007393), as described previously^41^. GAPDH and β-actin mRNA was also analyzed to normalize differences in sample uptake. The quantitative (q)PCR were performed using IQ2 SYBR Green Fast qPCR System Master Mix (Bio-Genesis Technologies, Taiwan) and CFX96 Real-Time PCR Detection System (Bio-Rad, CA), as described previously^42^.

### Western blotting

HepG2 cells and Hepa-1c1c7, c4 and c12 cells were seeded at 8 × 10^5^ cells/6-cm dish overnight. Afterwards, cells were cultured with test compounds for appropriate time periods. At the end of the desired treatment times, cell lysates were prepared, and Western blots were performed as described previously^43^. The blots were cut and the area of blot corresponding to CYP1A1 or GAPDH was selected for hybridization individually and shown in the figures (Supplementary Information).

### Reporter plasmids and reporter activity assay

The pAhRDtkLuc3 comprises three AHRE motifs linked to the HSV-TK minimum promoter^44,45^ in the pGL3-basic vector. The RSV-lacZ plasmid contains a *lacZ* gene-encoded β-galactosidase, with a Rouse sarcoma virus (RSV) LTR as the promoter.

MCF cells, HepG2 cells and Hepa-1c1c7 cells were subcultured at 6 × 10^4^, 6 × 10^4^, and 2.5 × 10^4^ cells/well respectively, in a 24-well plate overnight. Afterwards, the luciferase reporter plasmid and RSV-*lac*Z plasmids were transfected into cells using the liposome for 6 h, followed by treatment with the test compounds as described previously^34^. Cell lysates were harvested at the appropriate time points after treatment with test compounds and were respectively assayed for both luciferase and β-galactosidase activities using Britelite (PerkinElmer) and the Galacto-Star System (Tropix, Bedford, MA) as described previously^34,41^. Transcription activity of the promoter was indicated by luciferase activity, and β-galactosidase activity of RSV-*lac*Z was used to normalize the luciferase activity.

### Immunocellular fluorescence staining

To analyze in situ CYP1A1 expression, Hepa-1c1c7 cells were seeded at 3 × 10^5^ cells/well in 6-well plates with microscope cover glasses in the well for more than 14 h and then treated with test compounds, followed by washing with phosphate-buffered saline (PBS) and being fixed with ethanol, as described previously^46^. The detection of the in situ CYP1A1 expression by fluorescence emission in Hepa-1c1c7 cells was described previously^41^.

To monitor the translocation of the AhR into nucleus, Hepa-1c1c7 cells were seeded at 2 × 10^5^ cells/well in 6-well plates with microscope cover glasses in the well overnight, and then were cultured with test compounds, followed by washing with PBS and being fixed with 4% formaldehyde in PBS. The detailed procedures to fix the cells and probe the AhR by fluorescence were performed as described previously^41,46^.

### Statistical analysis

Data are representative of at least three independent experiments under identical conditions and are expressed as the mean ± standard deviation (SD). Differences among the data of the control and further treatments with various compounds were analyzed by Student’s *t-*test. The statistical probability (p) was expressed as *p < 0.05, **p < 0.01, and ***p < 0.001. Means were considered significantly different at p < 0.05.

## Supplementary Information

Supplementary Information

## Abbreviations

AhR: Aryl hydrocarbon receptor
AHRE: Aryl hydrocarbon response element
CPA: Cyproterone acetate
CYP: Cytochrome P450
