# Supplementary material for: Cyproterone acetate acts as a disruptor of the aryl hydrocarbon receptor
Source: Sci Rep. 2021 Mar 9;11:5457. doi: 10.1038/s41598-021-84769-7 (PMC7943802; doi:10.1038/s41598-021-84769-7)

Full Title

Cyproterone acetate acts as a disruptor of the aryl hydrocarbon receptor

Authors

Chih-Shou Chen [1], Guan-Lun Gao [2, 3], Dong-Ru Ho [1], Chih-Yi Lin [2], Yu-Ting Chou [2], Shan-Chun Chen [2], Min-Cong Huang [1], Wen-Ya Kao [2], Jyan-Gwo Joseph Su [2, *]

Affiliations

[1]Division of Urology, Department of Surgery,
Chang Gung Memorial Hospital, Chiayi, Taiwan, ROC

[2]Department of Biochemical Science and Technology, National Chiayi University,
Chiayi 60004, Taiwan, ROC

[3]Department of Biological Resources, National Chiayi University, Chiayi, 60004, Taiwan, ROC

Fig. 3a.

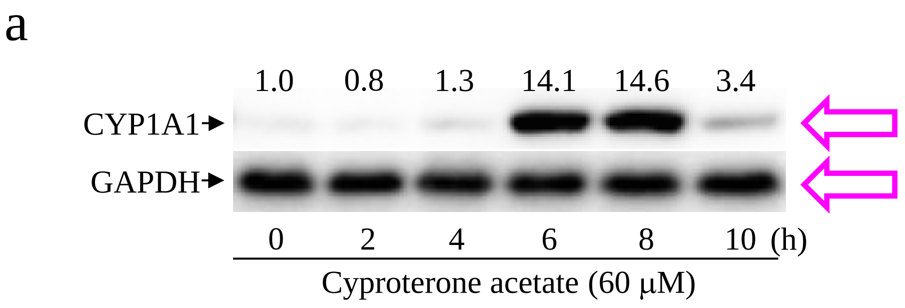
a
1.0 0.8 1.3 14.1 14.6 3.4
CYP1A1
GAPDH
0 2 4 6 8 10 (h)
Cyproterone acetate (60 µM)

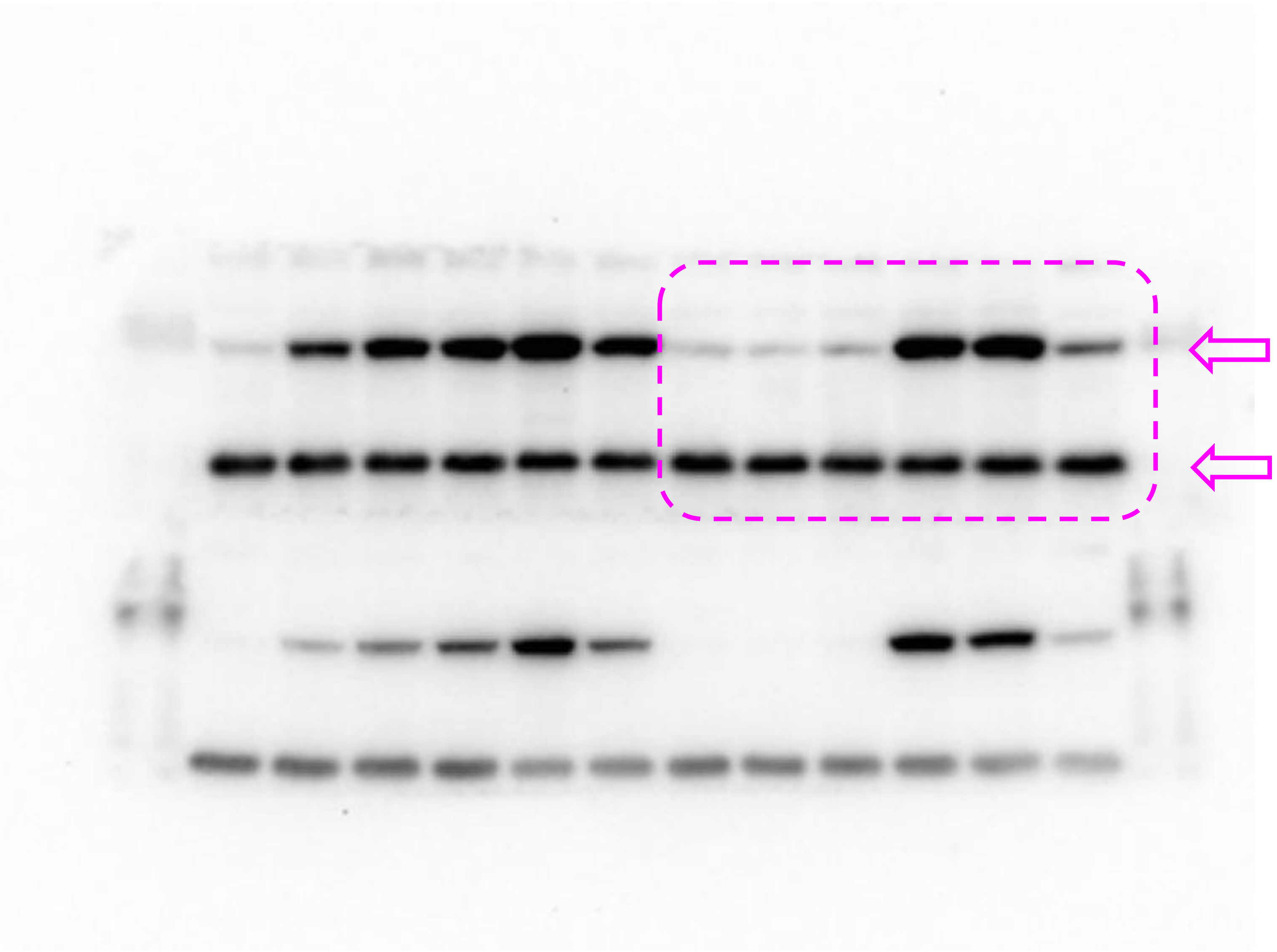

Fig. 3b.

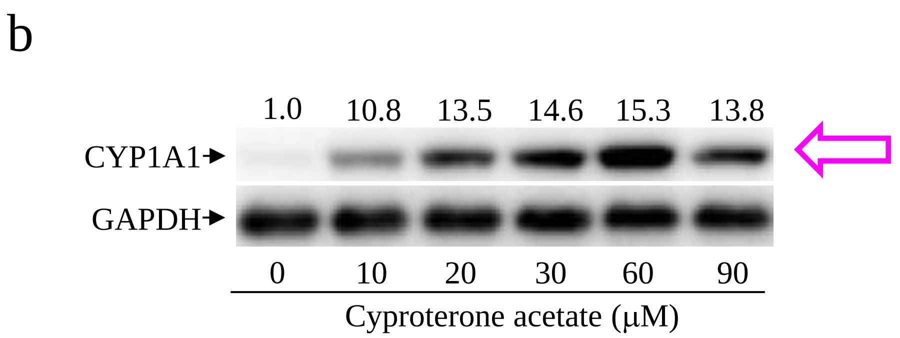
b
1.0
10.8
13.5
14.6
15.3
13.8
CYP1A1
GAPDH
0
10
20
30
60
90
Cyproterone acetate (μM)

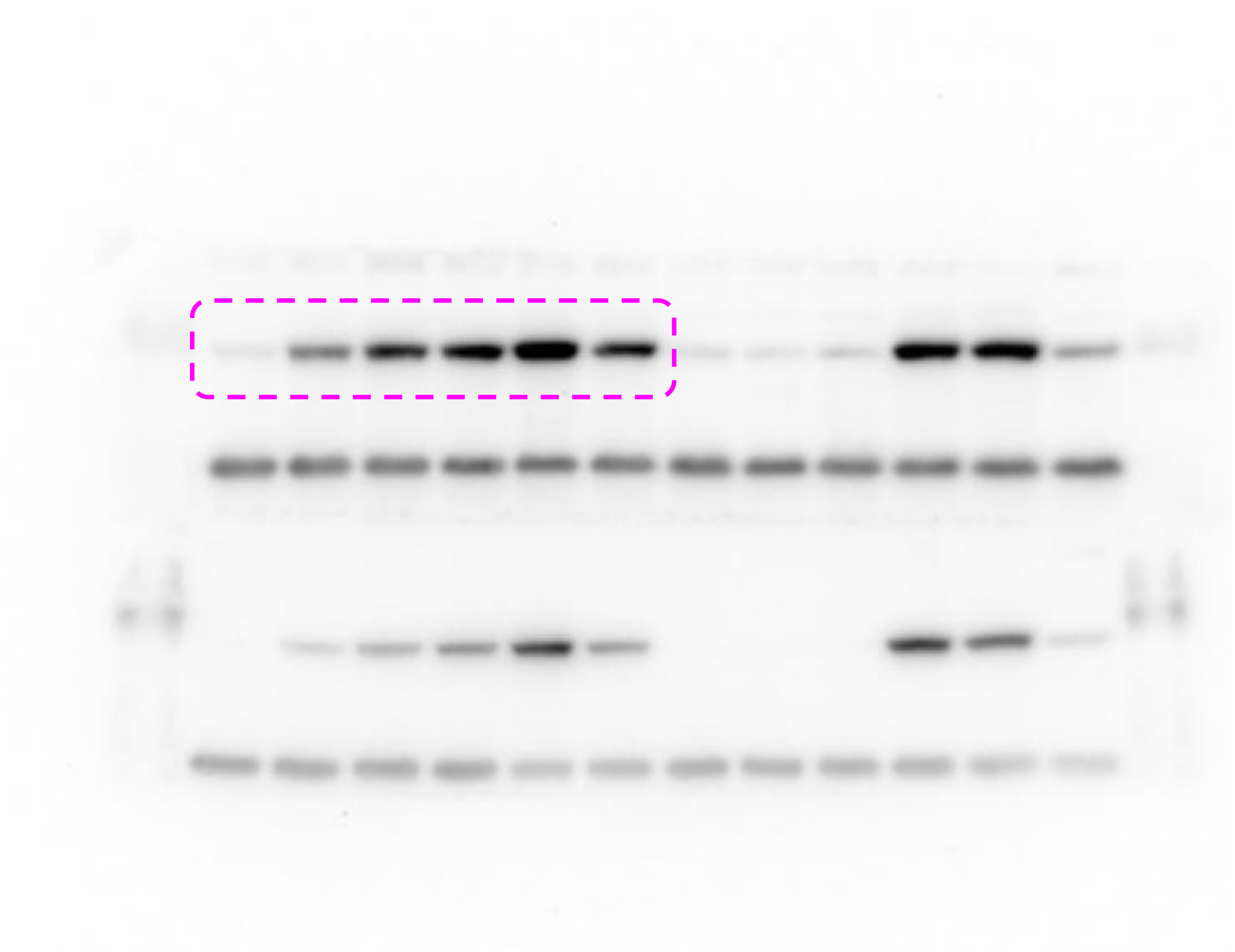

Fig. 3b (continued)..

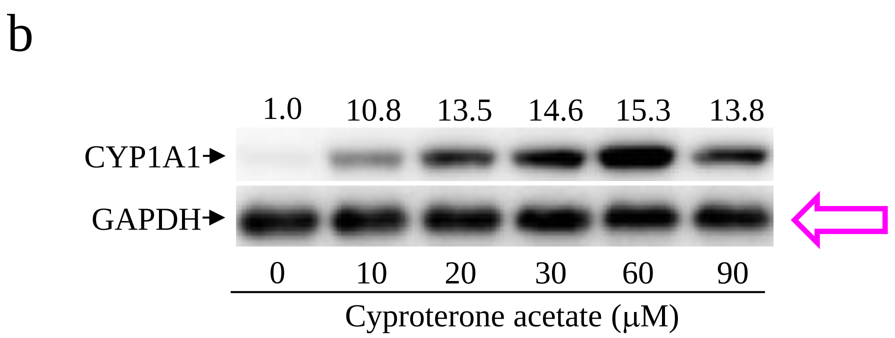

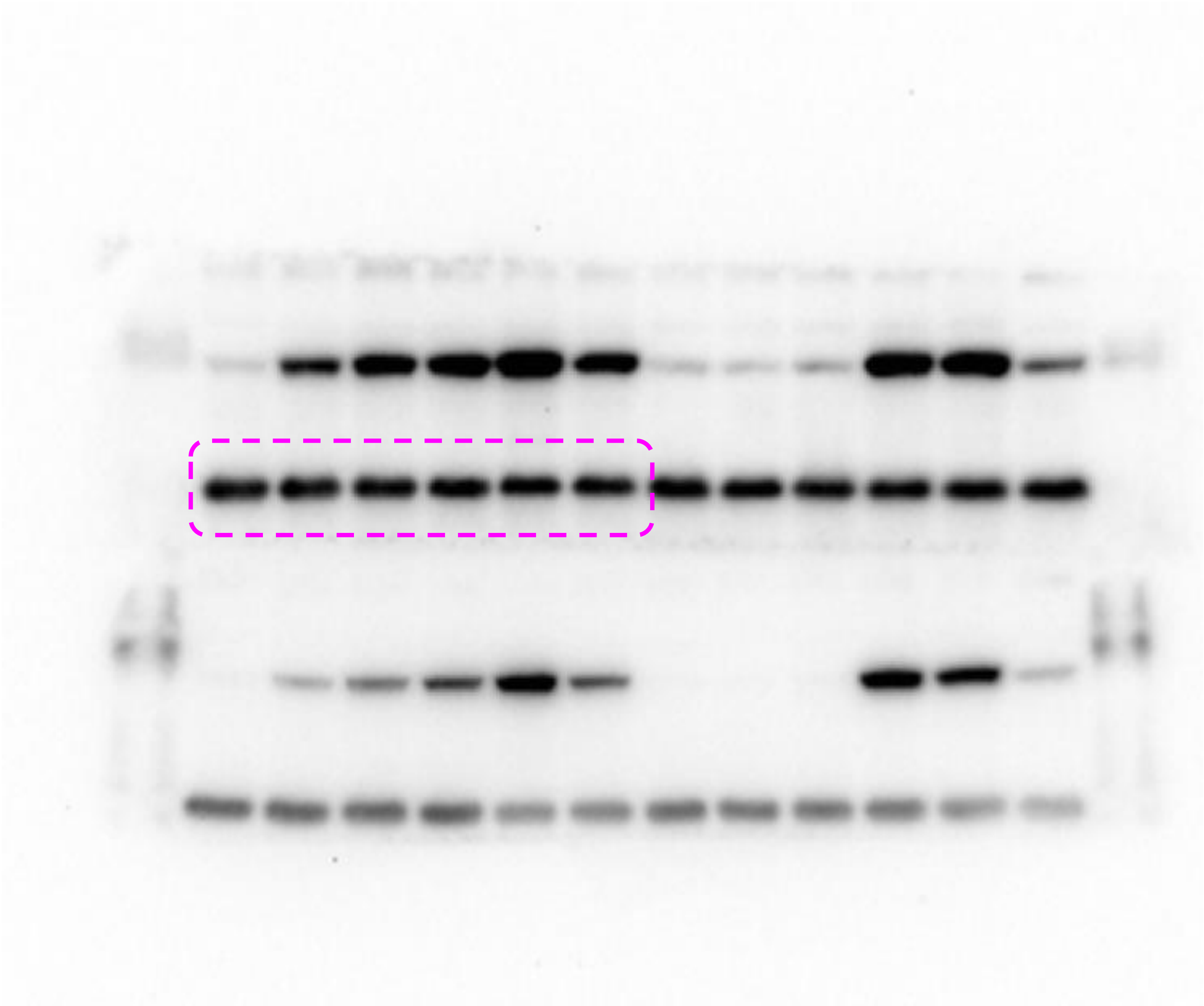

Fig. 5b.

b

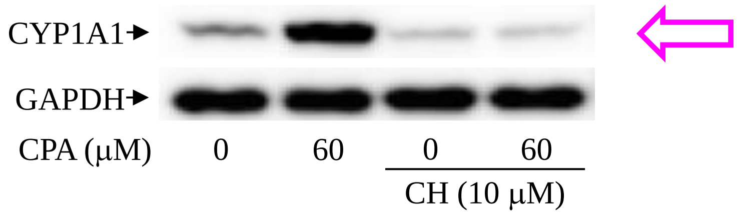

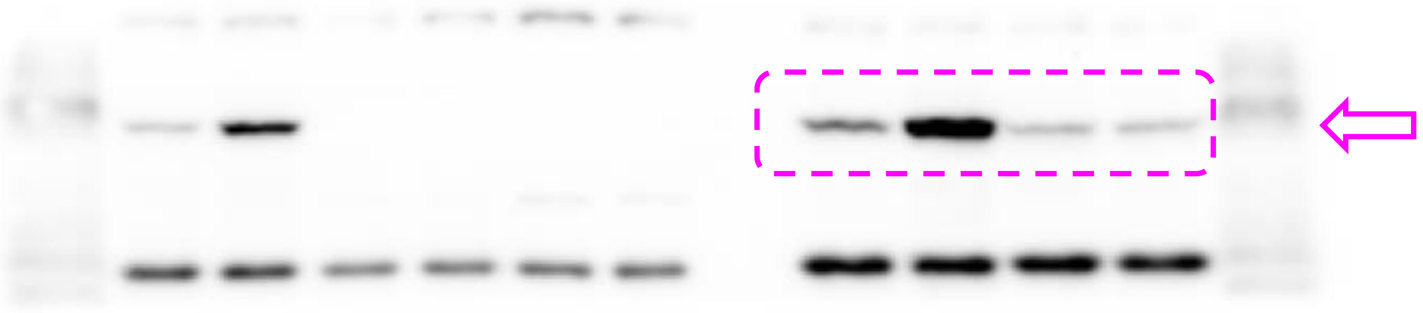

Fig. 5b (continued)..

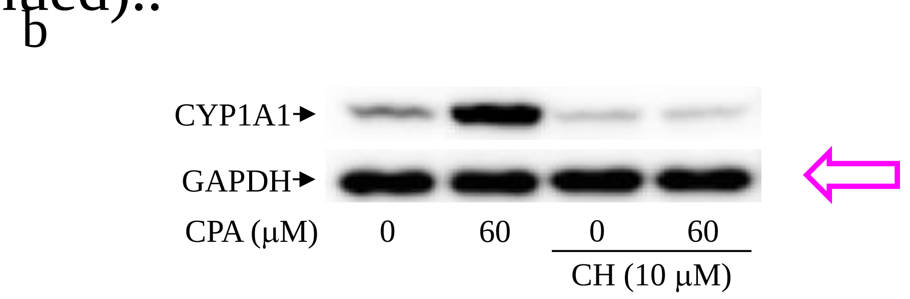

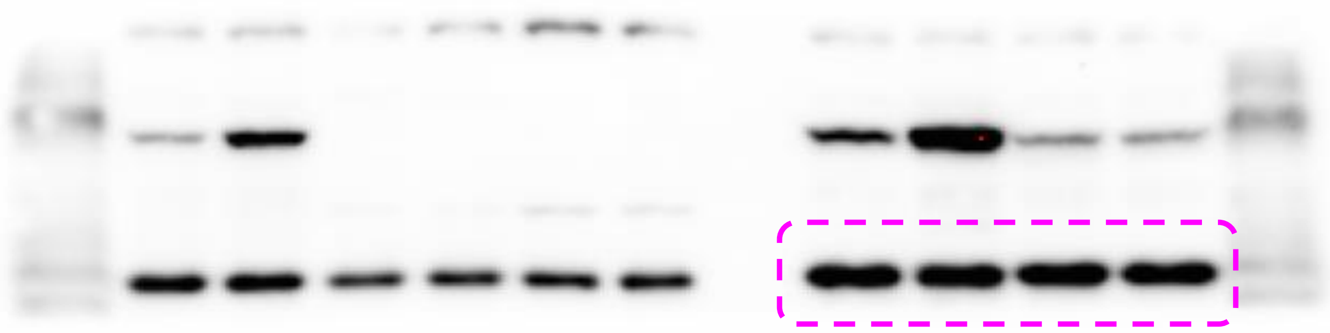

Fig. 5c.

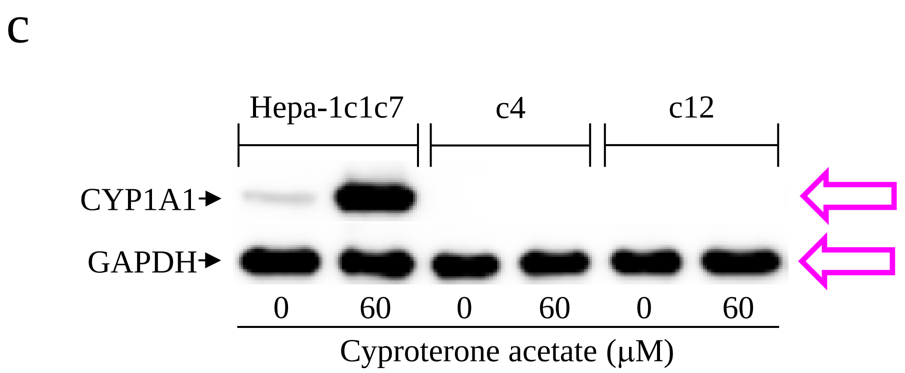

c
Hepa-1c1c7
c4
c12
CYP1A1
GAPDH
0
60
0
60
0
60
Cyproterone acetate (μM)

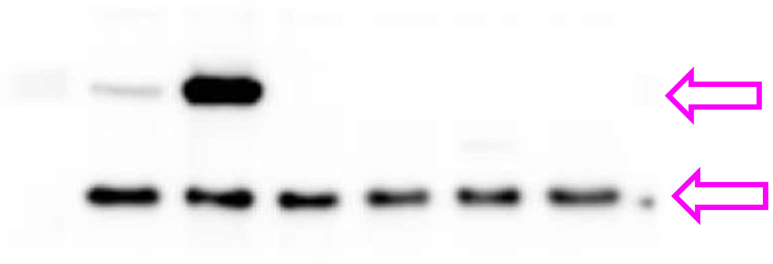

Fig. 5c (continued).

c

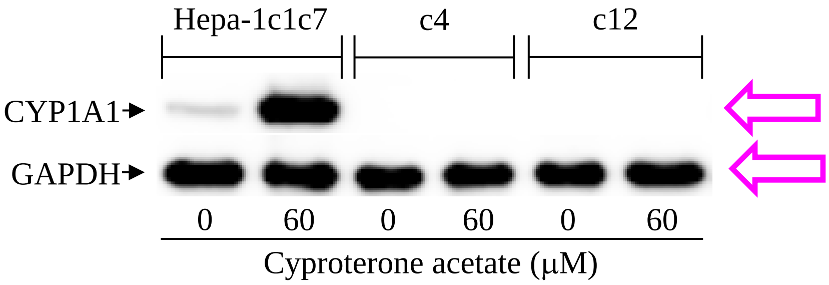

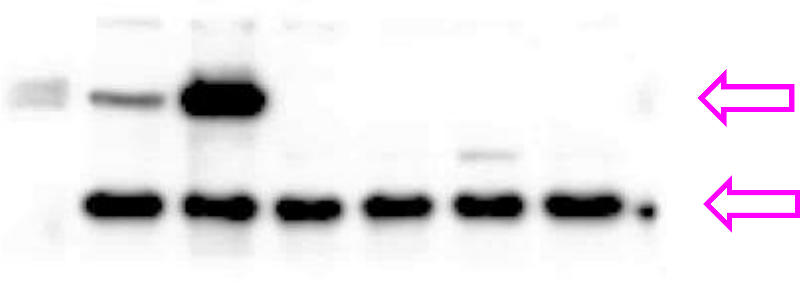

Fig. 7e.

e

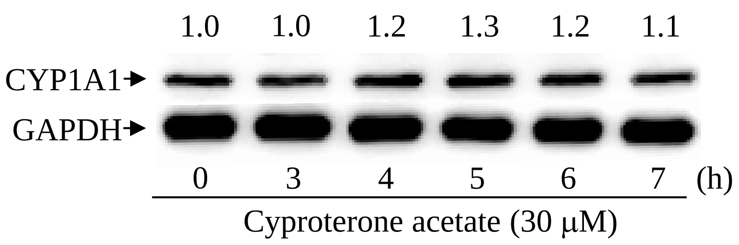
1.0 1.0 1.2 1.3 1.2 1.1
CYP1A1
GAPDH
0 3 4 5 6 7 (h)
Cyproterone acetate (30 μM)

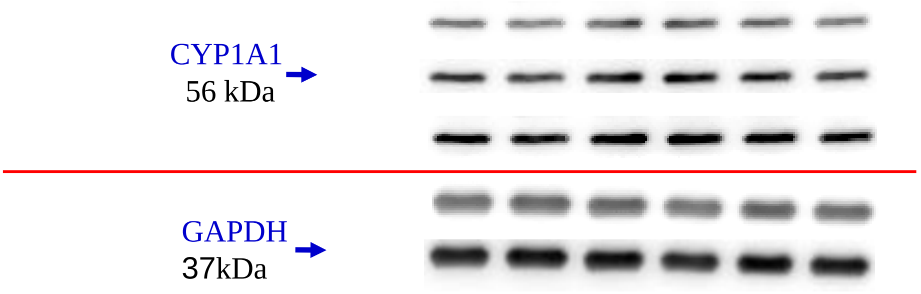
CYP1A1
56 kDa
GAPDH
37kDa

Fig. 7e (continued).
CYP1A1
p1

e

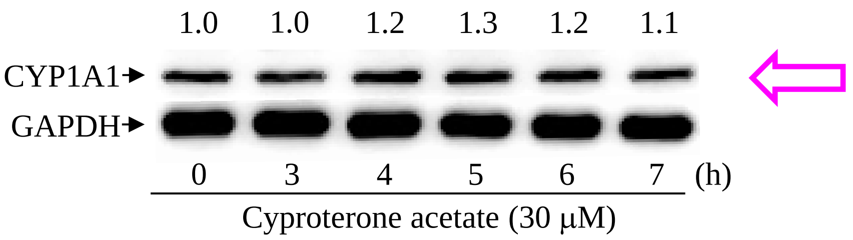

In order to decrease the volume of antibody, the blot was cut and the area of blot only corresponding to CYP1A1 or GAPDH was selected for hybridization individually.

The background of the original photograph is black, and it was turned to white.

TS_11_06_16_35_29.bmp

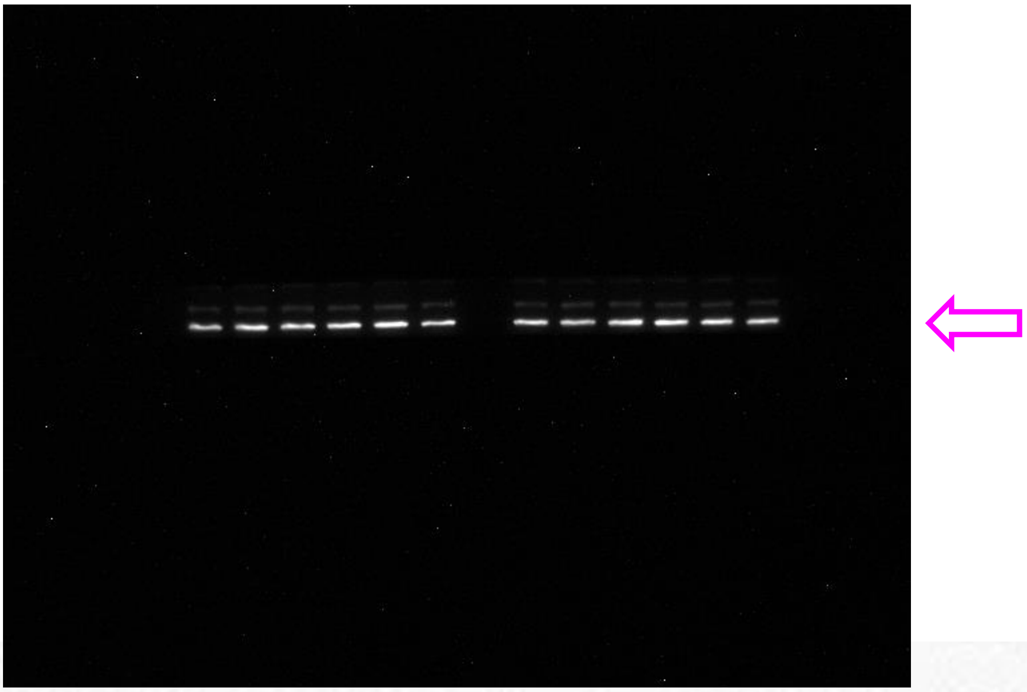

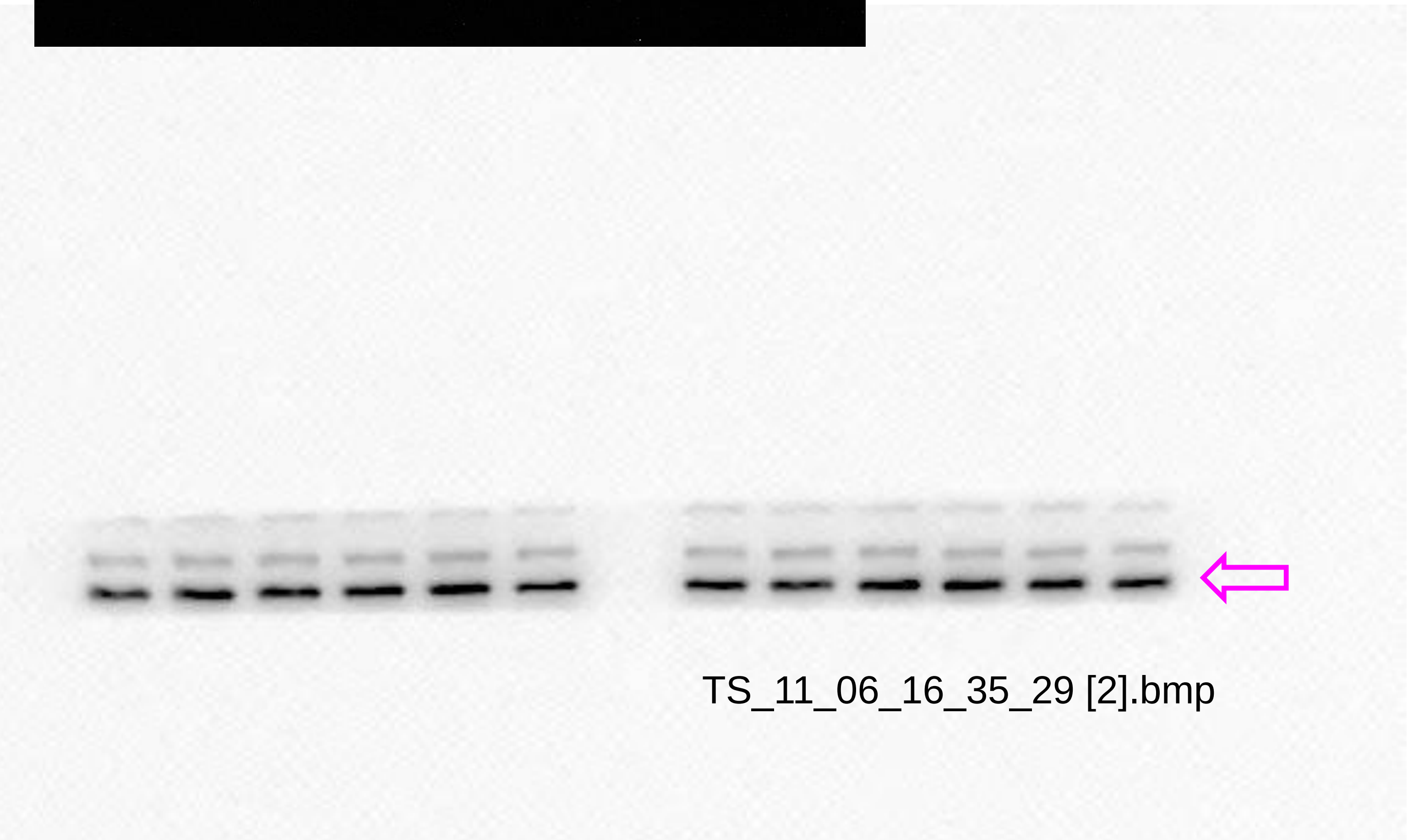

TS_11_06_16_35_29 [2].bmp

Fig. 7e (continued).
CYP1A1 e
p2

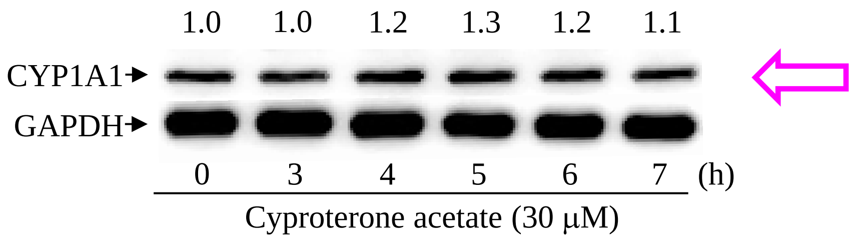

In order to decrease the volume of antibody, the blot was cut and the area of blot only corresponding to CYP1A1 or GAPDH was selected for hybridization individually.

The background of the original photograph is black, and it was turned to white.

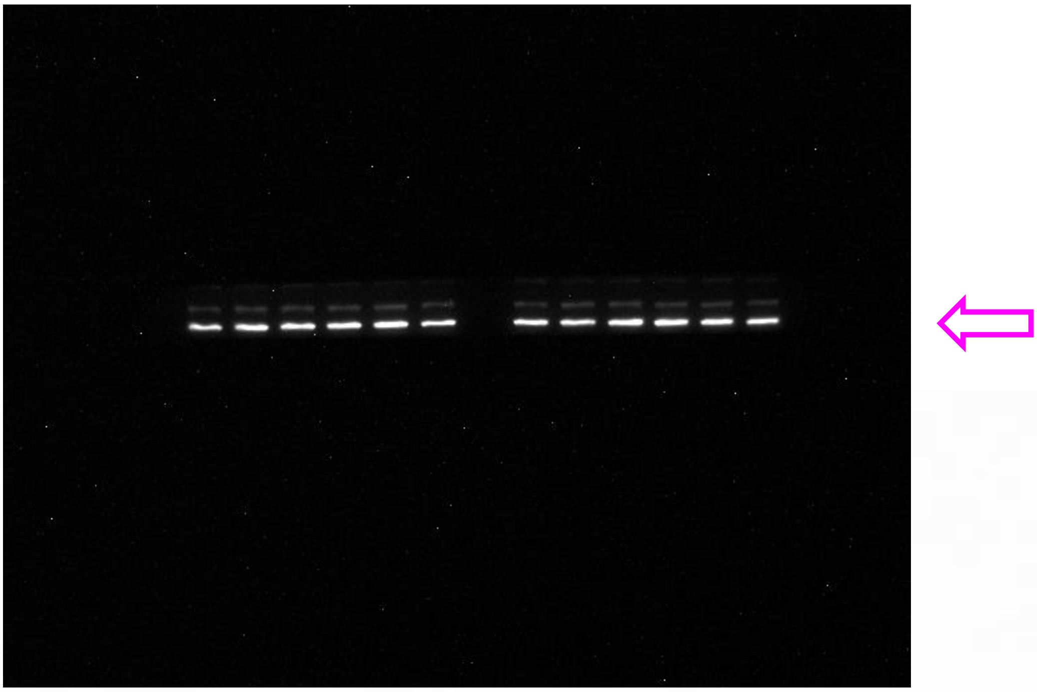

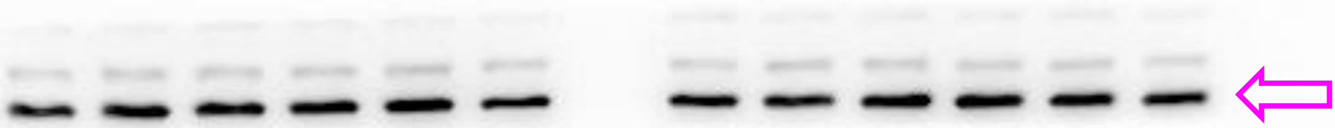

Fig. 7e (continued).

CYP1A1 e

p3

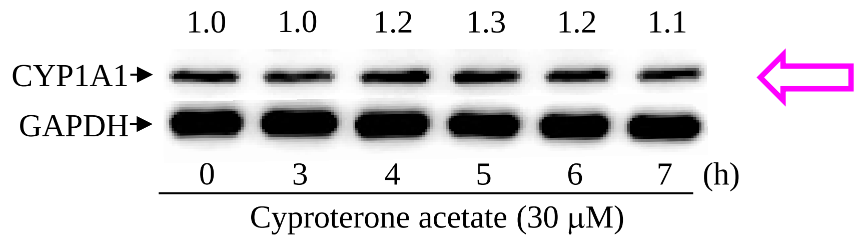

In order to decrease the volume of antibody, the blot was cut and the area of blot only corresponding to CYP1A1 or GAPDH was selected for hybridization individually.

The background of the original photograph is black, and it was turned to white.

TS_11_06_16_35_42.bmp

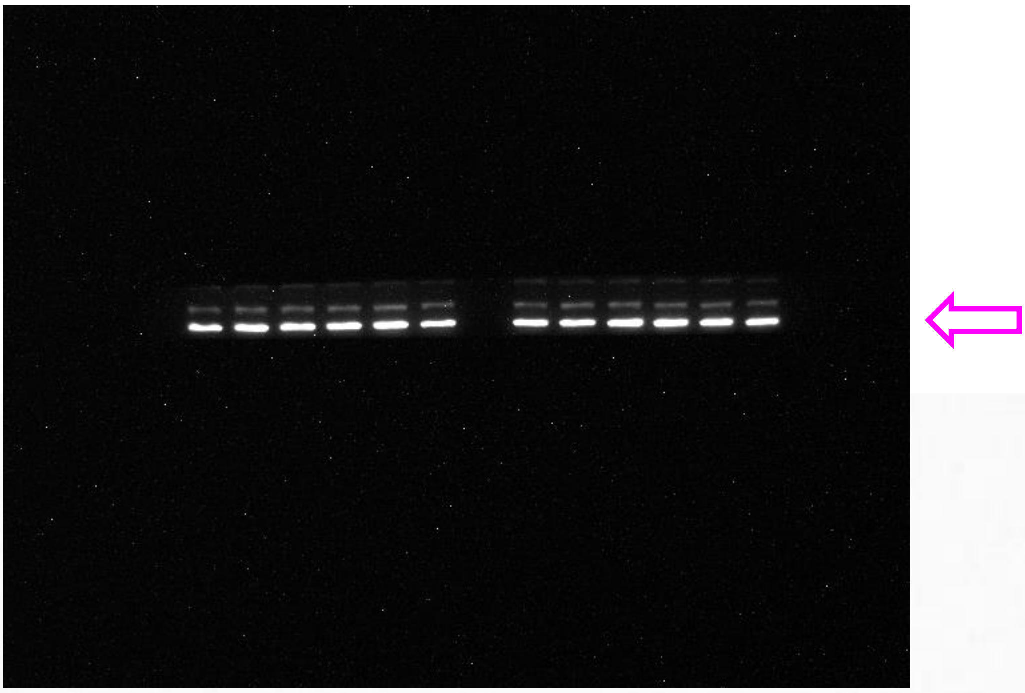

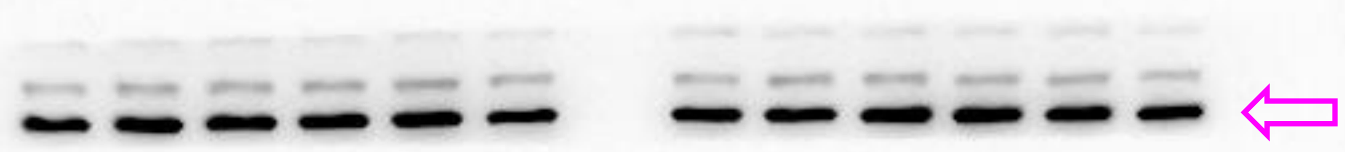

TS_11_06_16_35_42 [2].bmp

Fig. 7e (continued).
GAPDH

p1

e

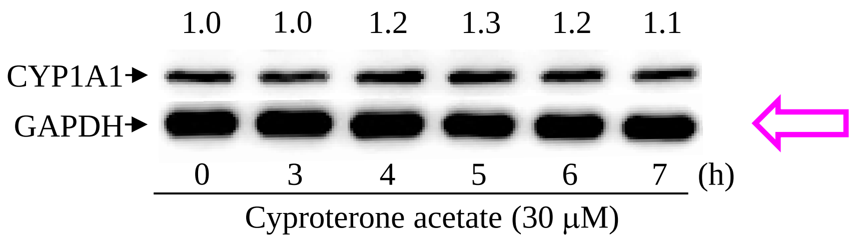

In order to decrease the volume of antibody, the blot was cut and the area of blot only corresponding to CYP1A1 or GAPDH was selected for hybridization individually.

The background of the original photograph is black, and it was turned to white.

TS_11_06_16_39_42.bmp

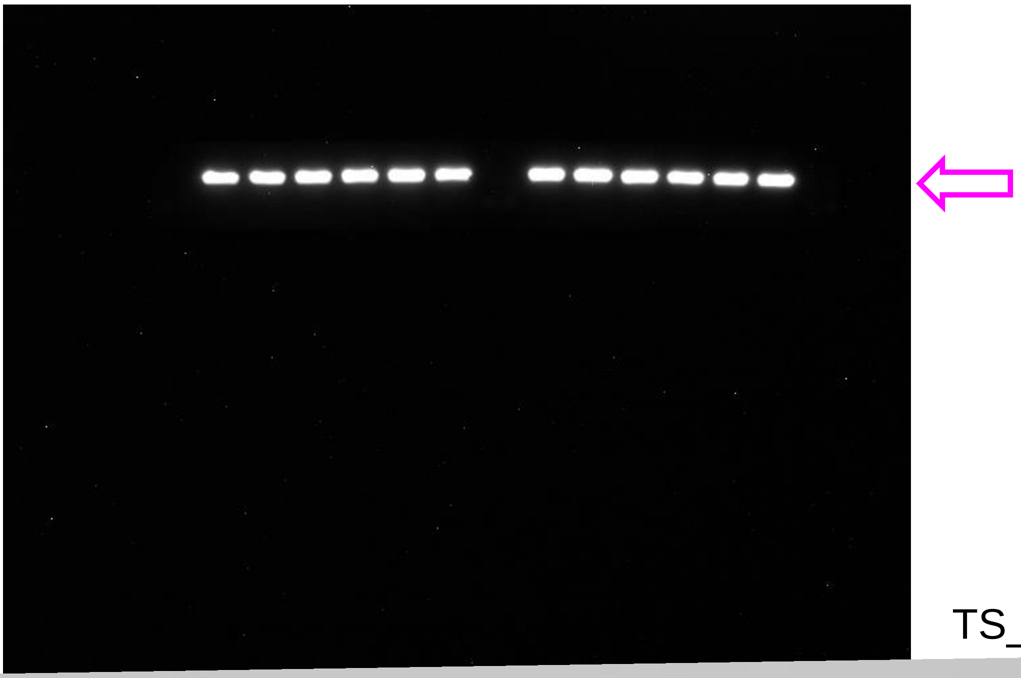

TS_11_06_16_39_42 [2].bmp

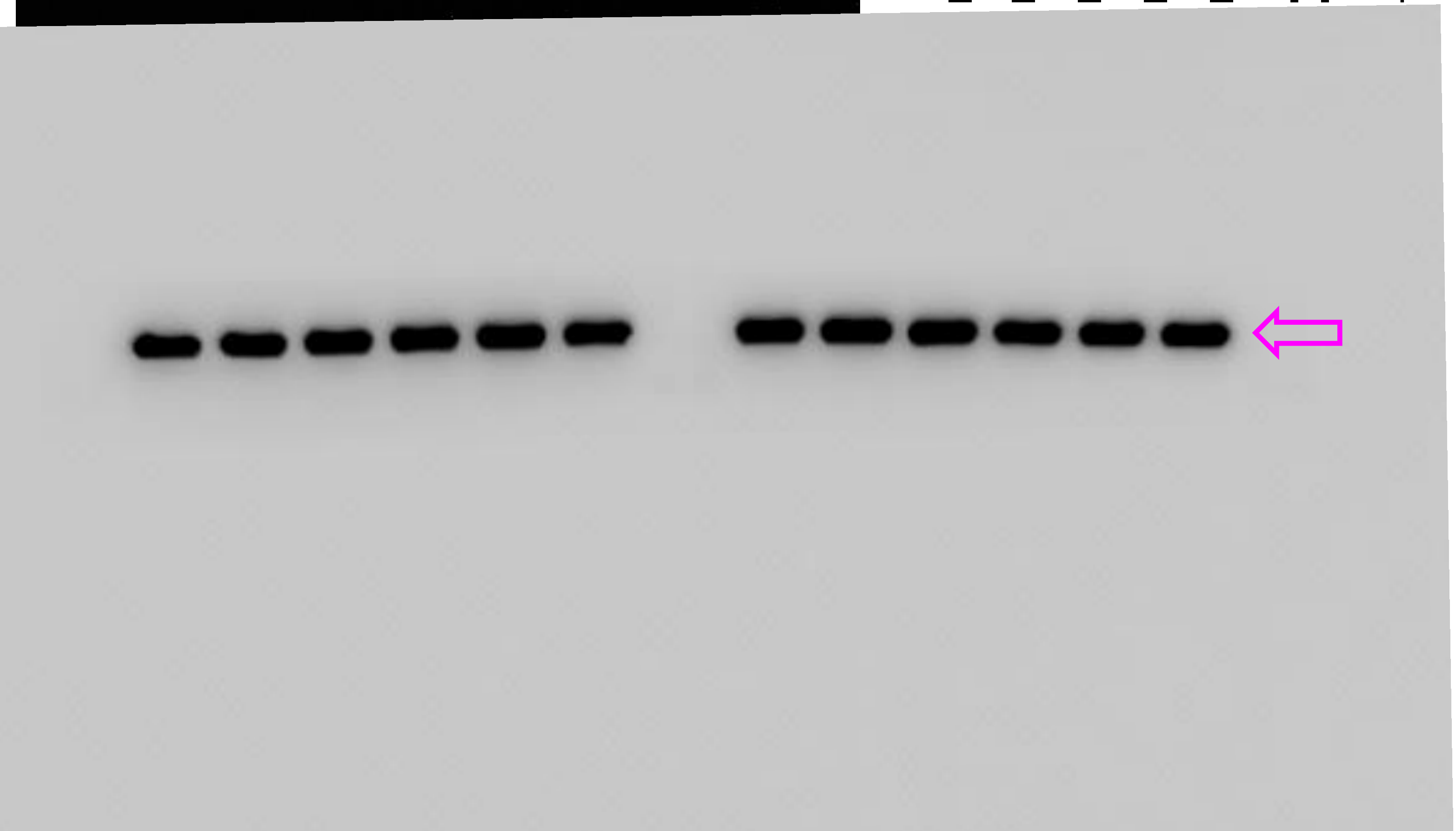

Fig. 7e (continued).
GAPDH
p2

e

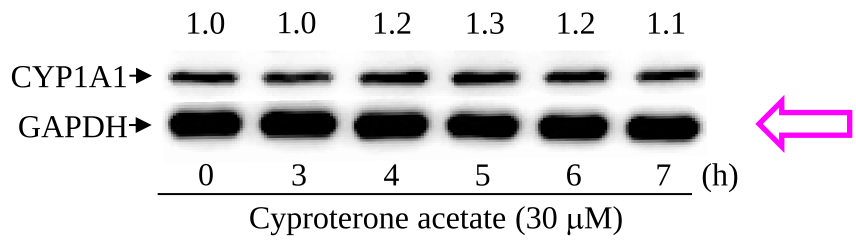

In order to decrease the volume of antibody, the blot was cut and the area of blot only corresponding to CYP1A1 or GAPDH was selected for hybridization individually.
The background of the original photograph is black, and it was turned to white.

TS_11_06_16_39_40.bmp

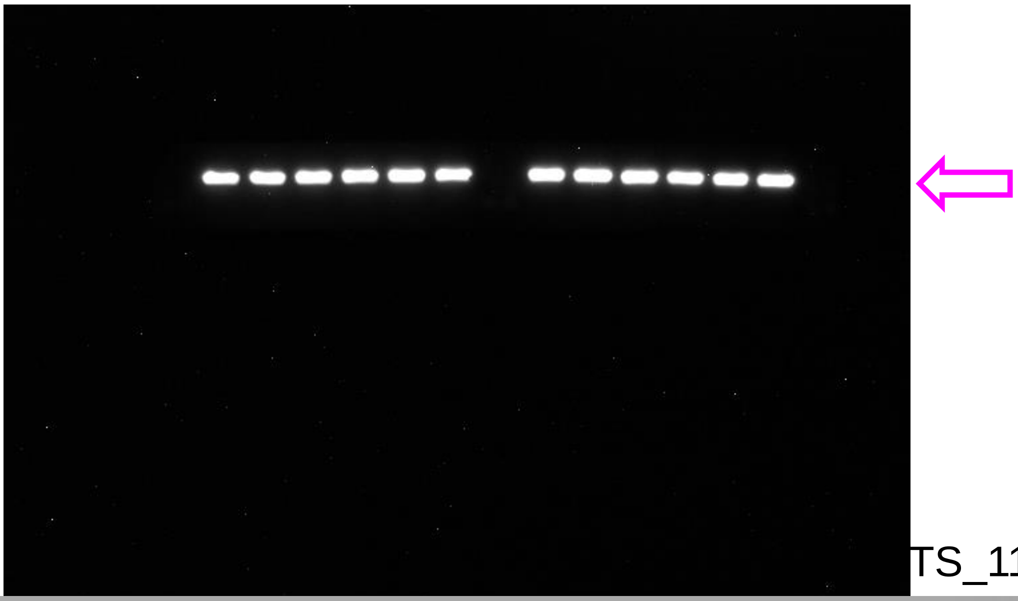

TS_11_06_16_39_40 [2].bmp

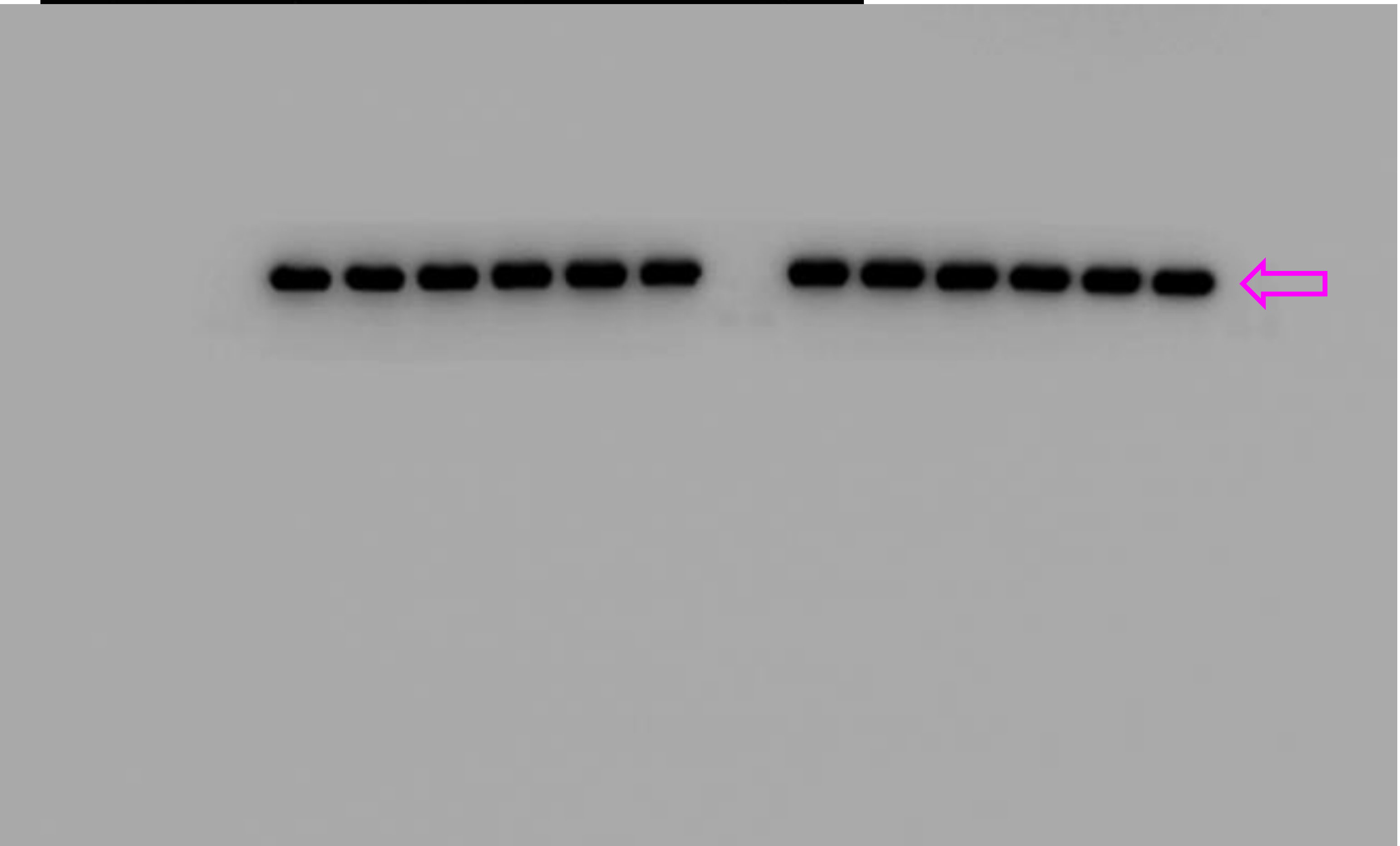

Fig. 7f.
CYP1A1
p1

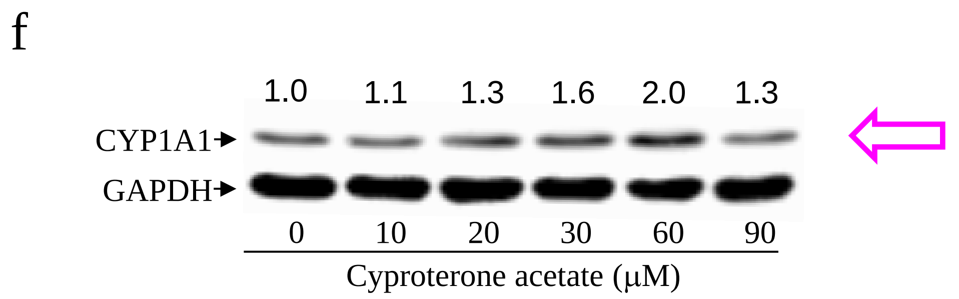

In order to decrease the volume of antibody, the blot was cut and the area of blot only corresponding to CYP1A1 or GAPDH was selected for hybridization individually.

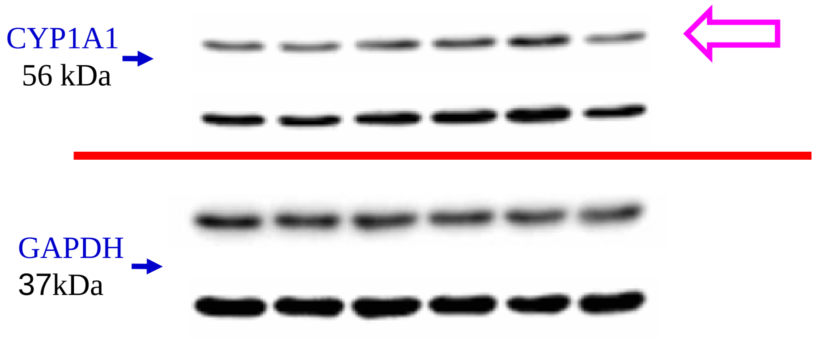

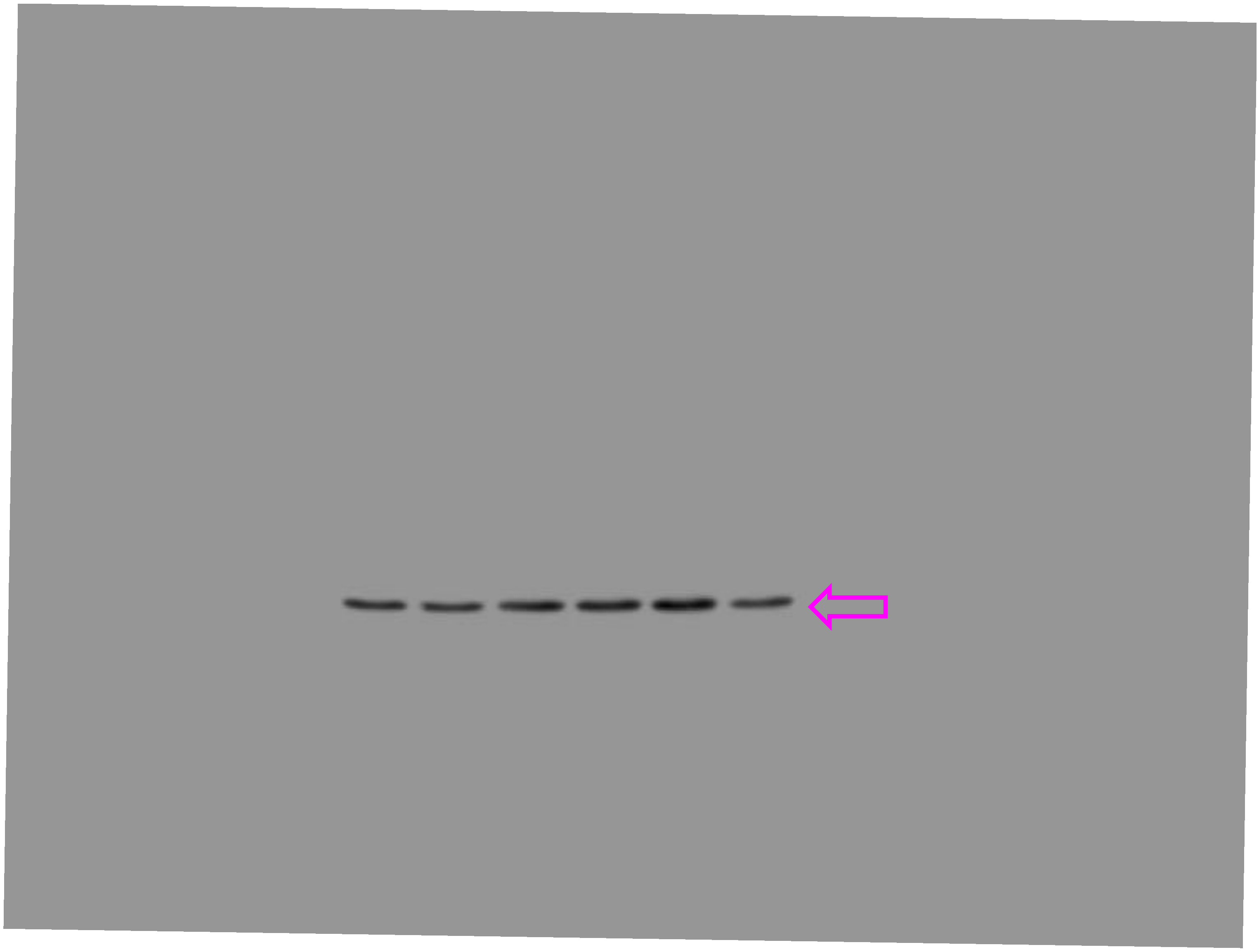

Fig. 7f (continued).
CYP1A1
p2

f

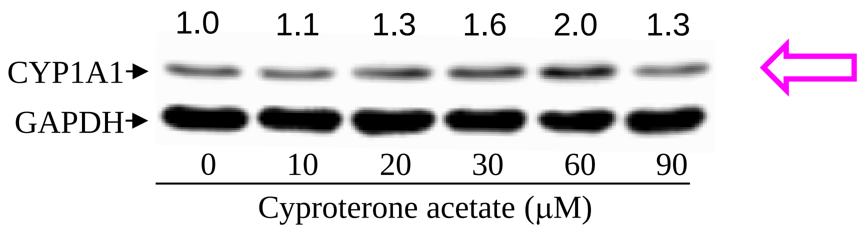

In order to decrease the volume of antibody, the blot was cut and the area of blot only corresponding to CYP1A1 or GAPDH was selected for hybridization individually.

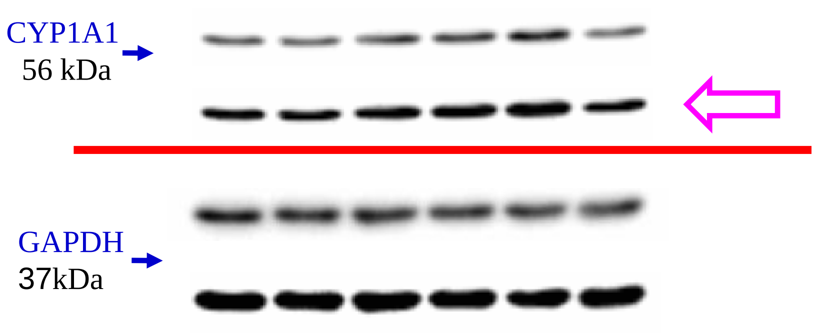

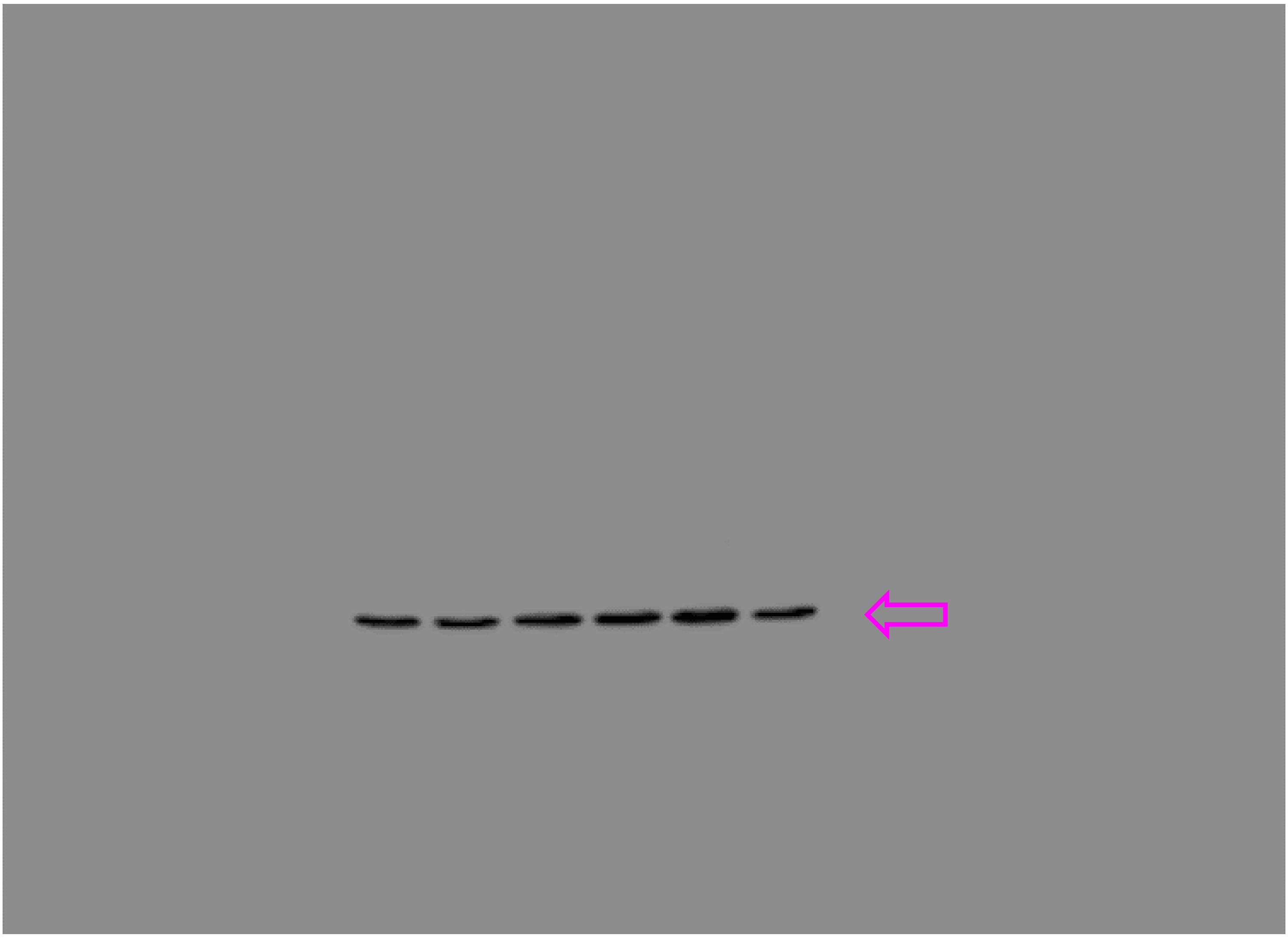

Fig. 7f (continued).
GAPDH
p1

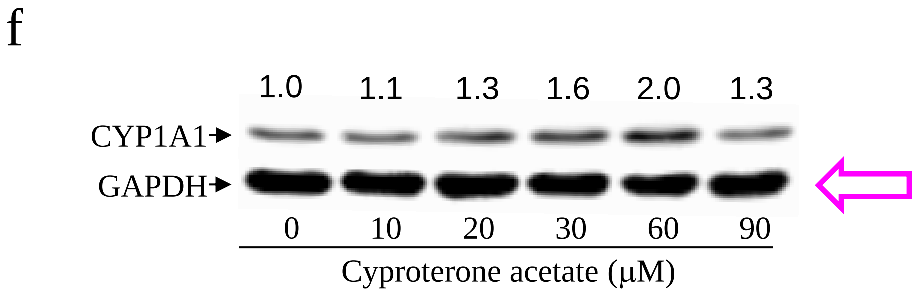

In order to decrease the volume of antibody, the blot was cut and the area of blot only corresponding to CYP1A1 or GAPDH was selected for hybridization individually.

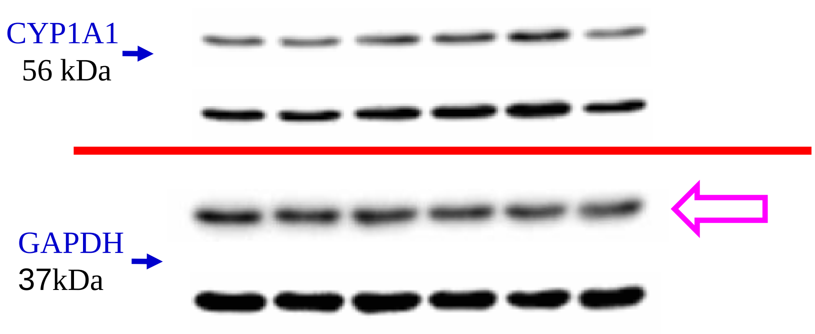

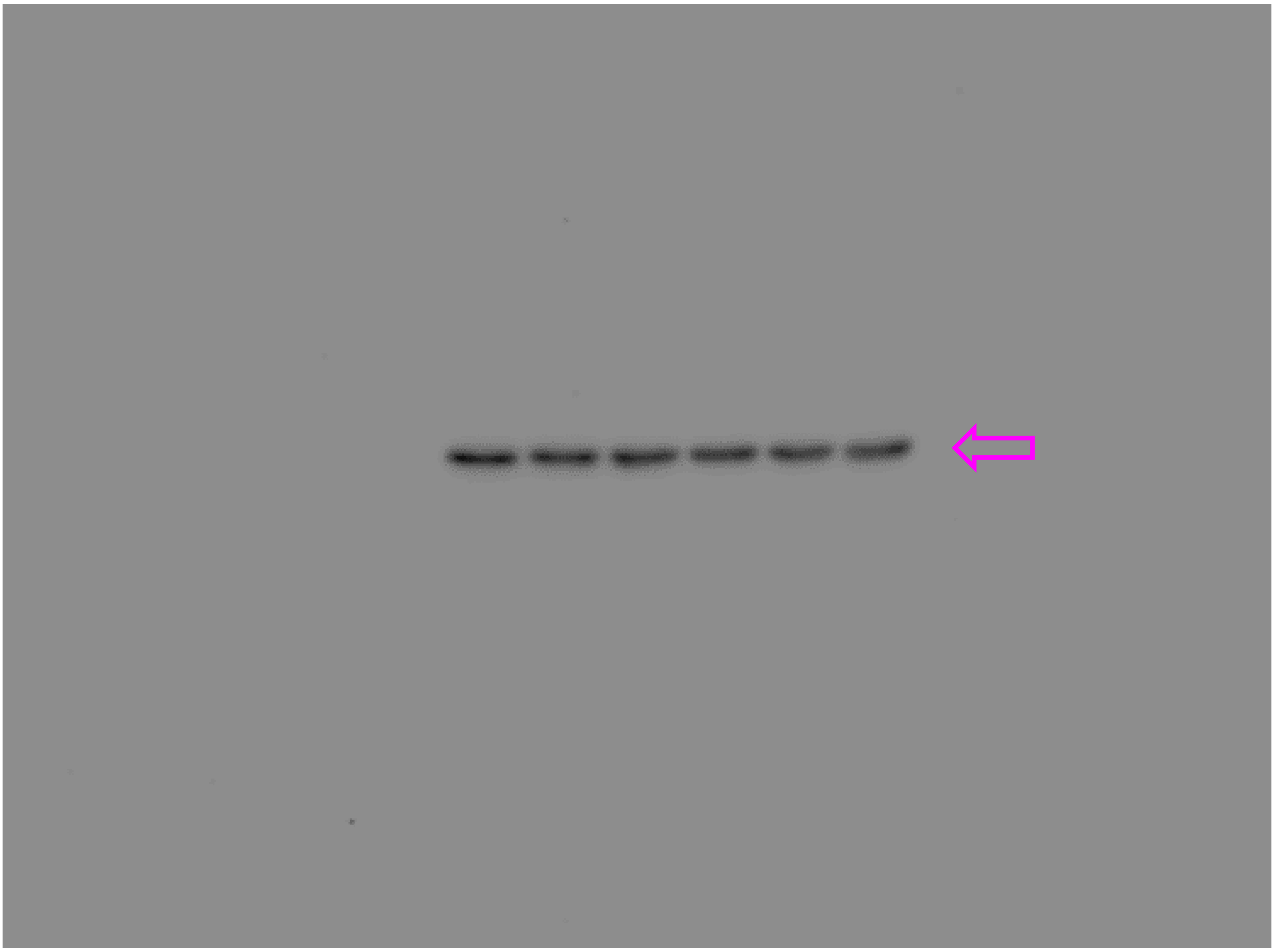

Fig. 7f (continued).
GAPDH
p2

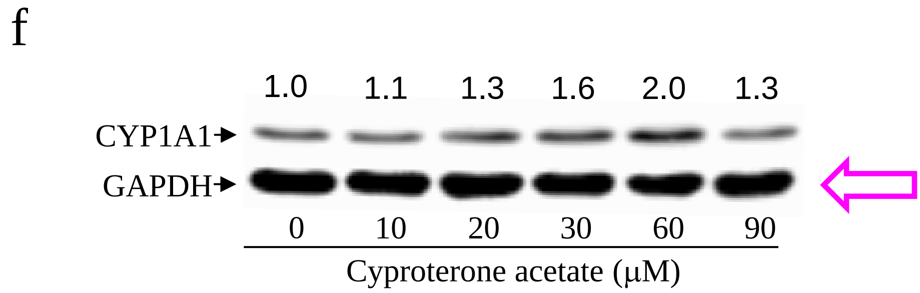

In order to decrease the volume of antibody, the blot was cut and the area of blot only corresponding to CYP1A1 or GAPDH was selected for hybridization individually.

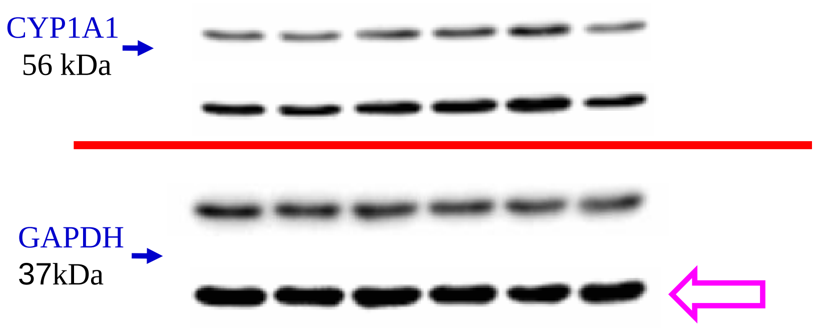

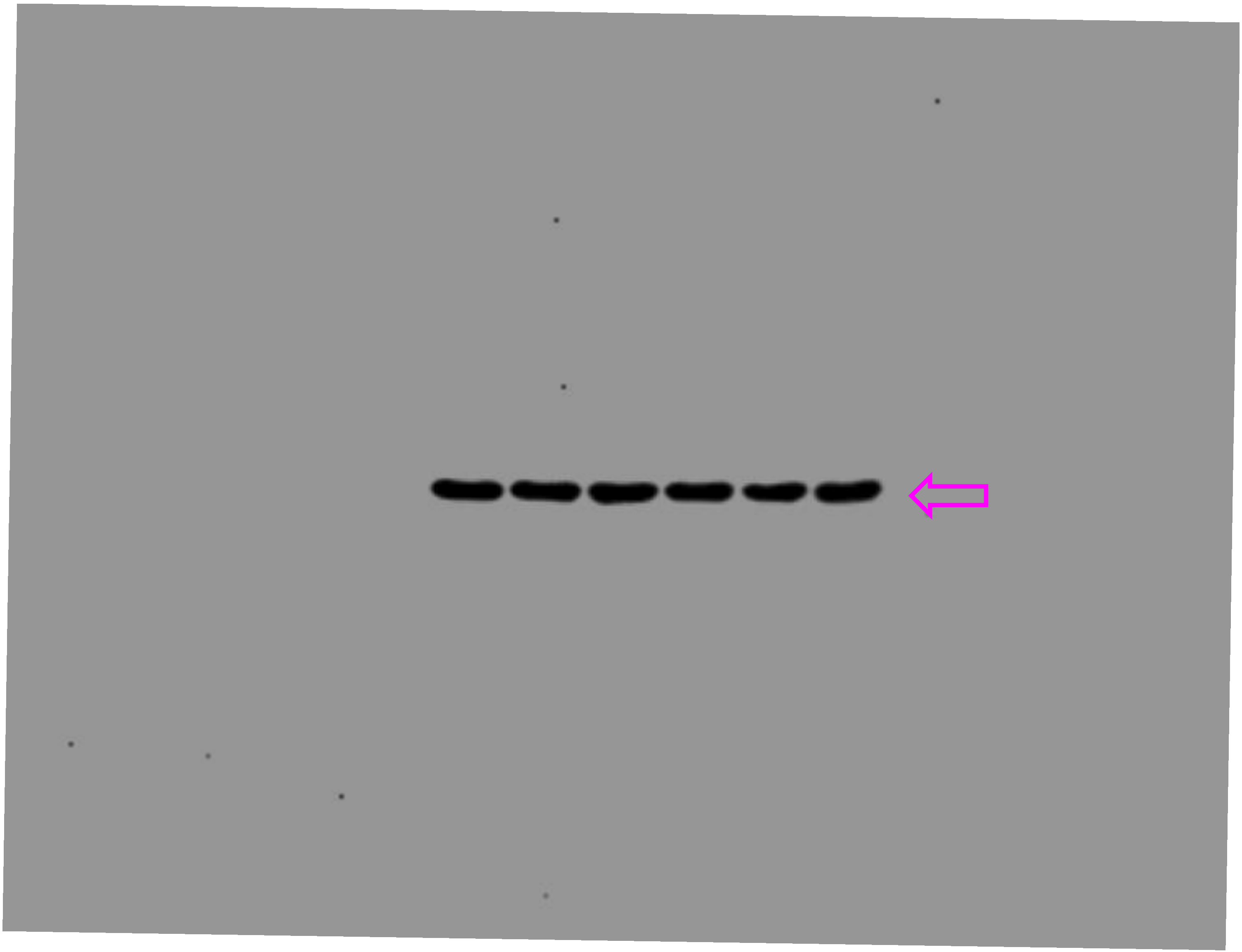

Fig. 7g.
CYP1A1 g
p1

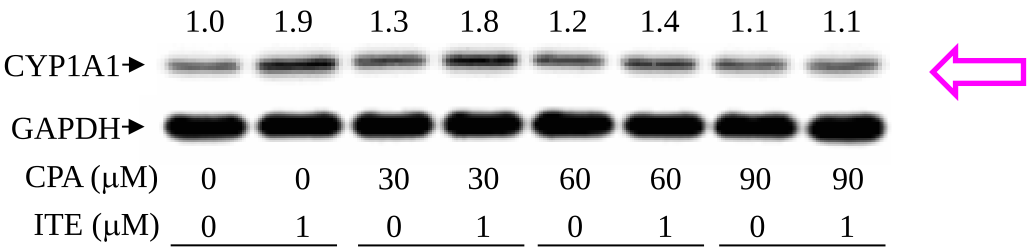

In order to decrease the volume of antibody, the blot was cut and the area of blot only corresponding to CYP1A1 or GAPDH was selected for hybridization individually.

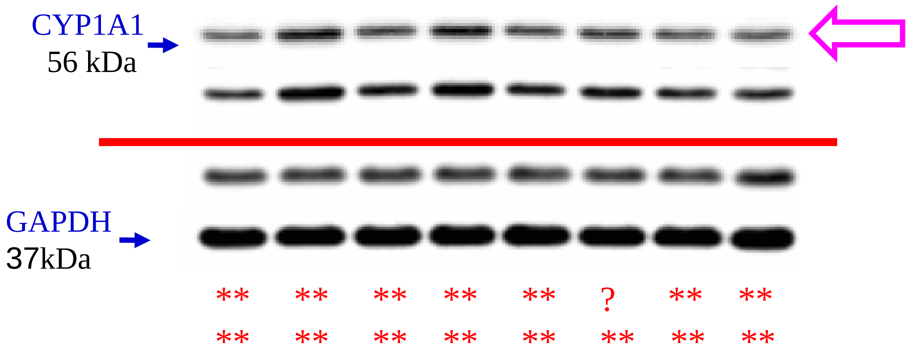

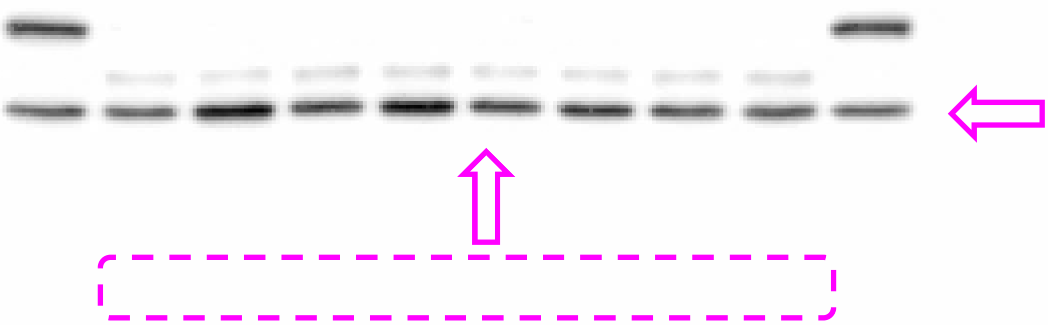

Fig. 7g (continued).
CYP1A1 g
p2

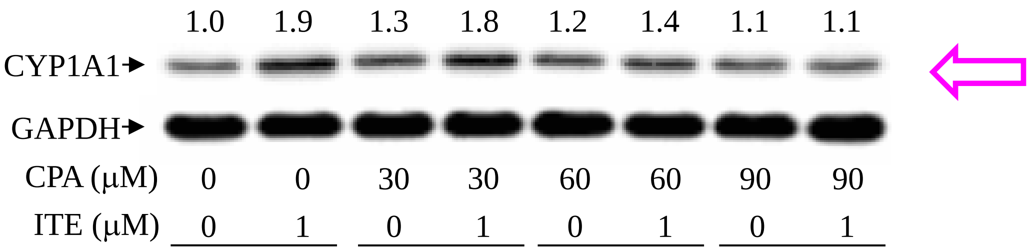

In order to decrease the volume of antibody, the blot was cut and the area of blot only corresponding to CYP1A1 or GAPDH was selected for hybridization individually.

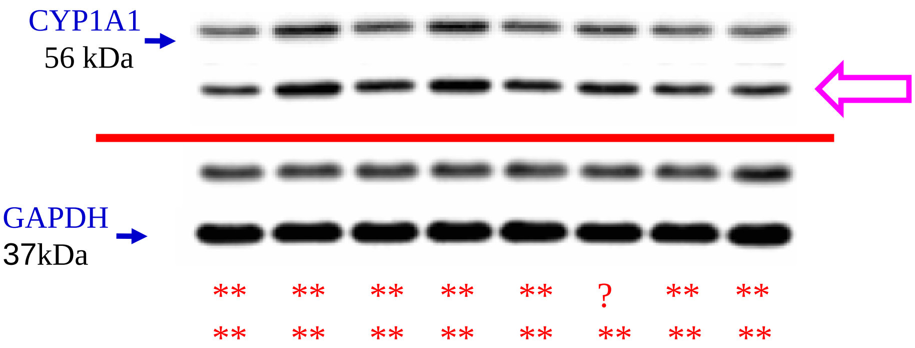

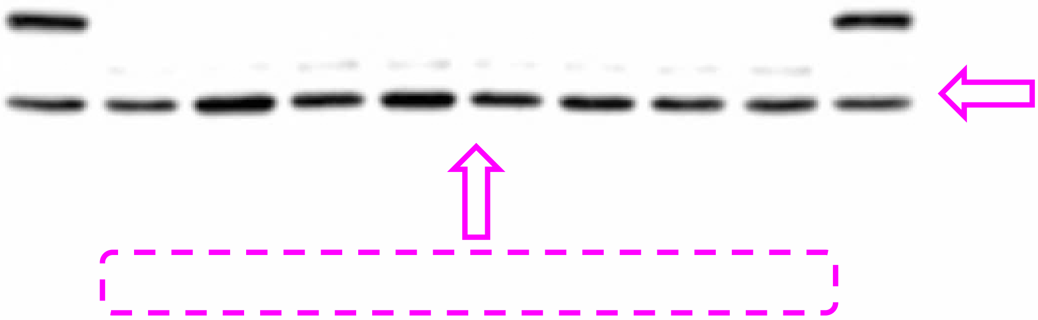

Fig. 7g (continued).

GAPDH g

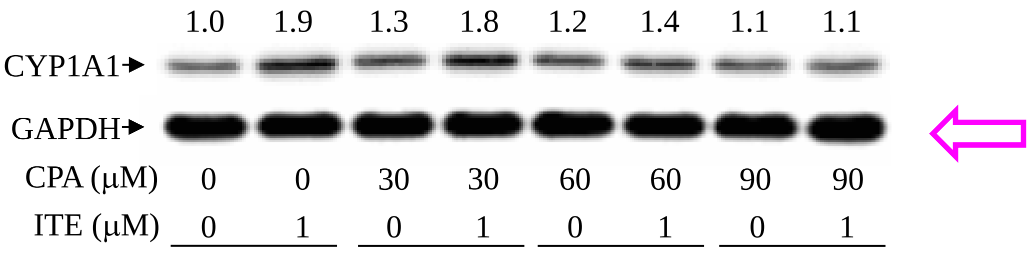

In order to decrease the volume of antibody, the blot was cut and the area of blot only corresponding to CYP1A1 or GAPDH was selected for hybridization individually.

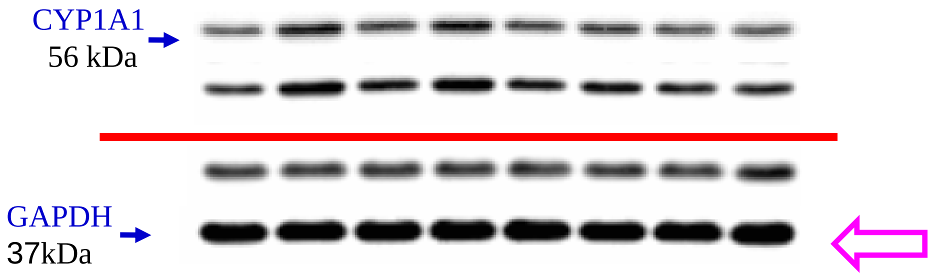

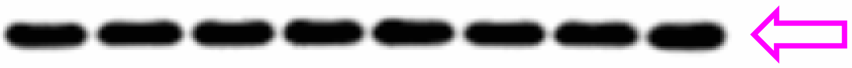

Fig. 7h.
CYP1A1
p1

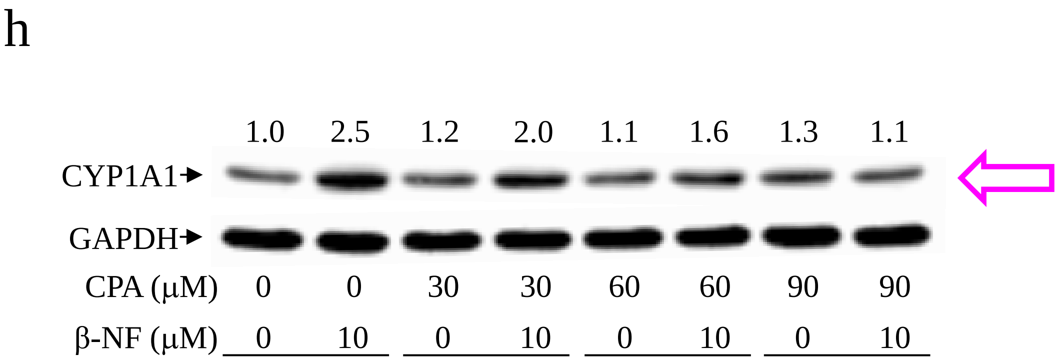

In order to decrease the volume of antibody, the blot was cut and the area of blot only w
corresponding to CYP1A1 or GAPDH was selected for hybridization individually.

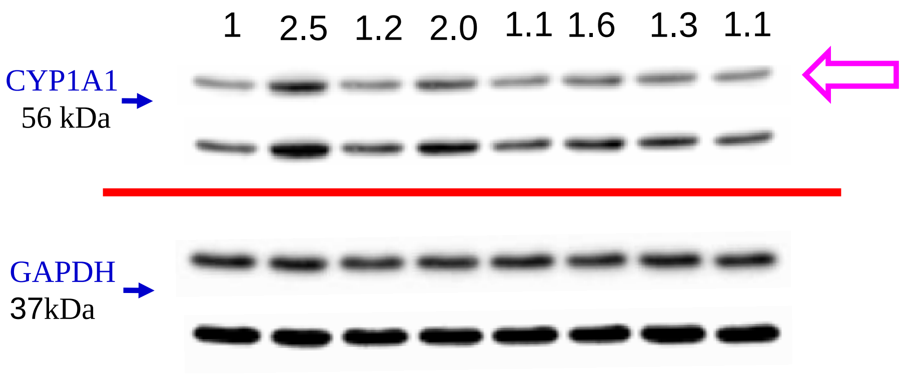

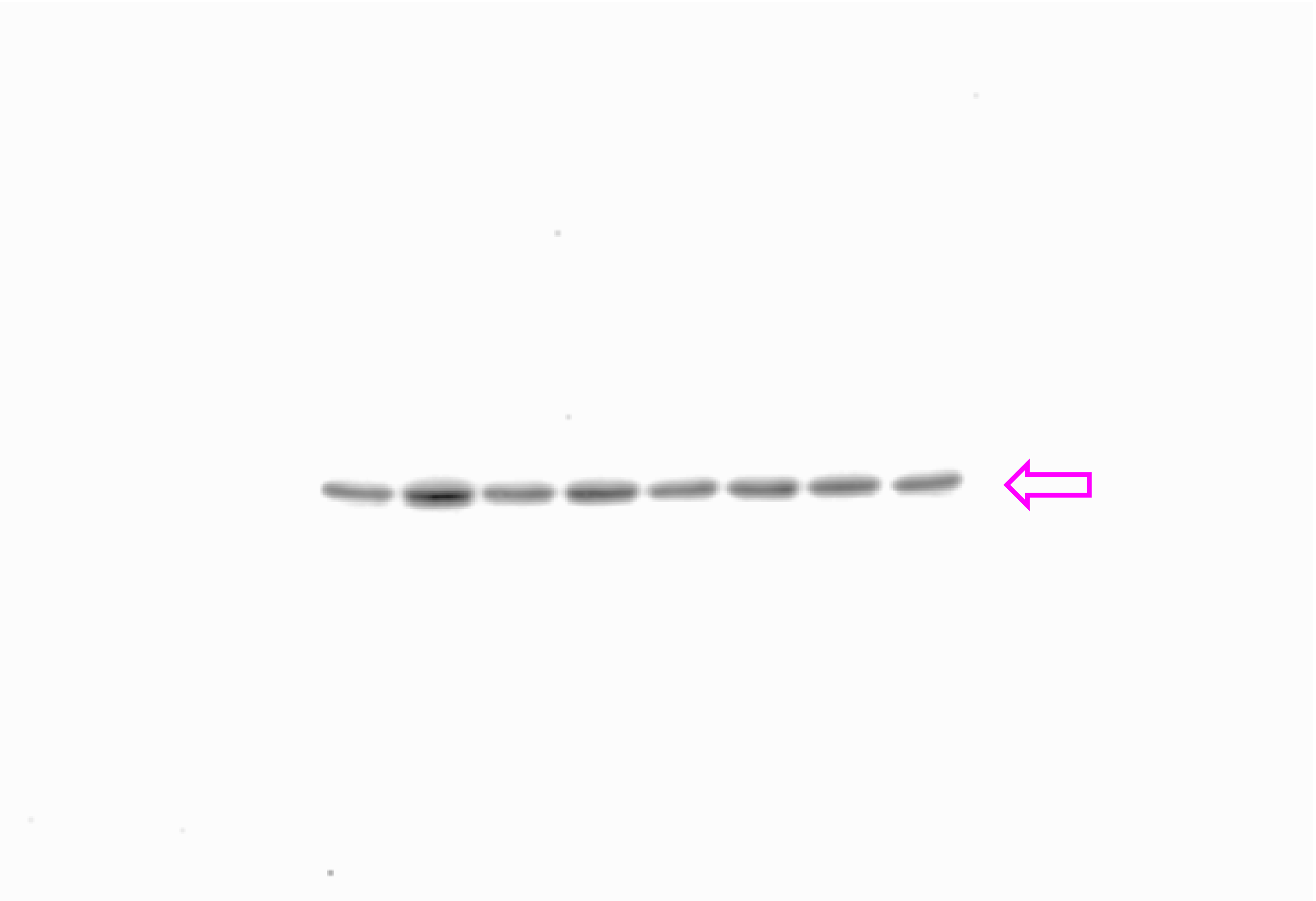

Fig. 7h (continued).
CYP1A1
p2

h

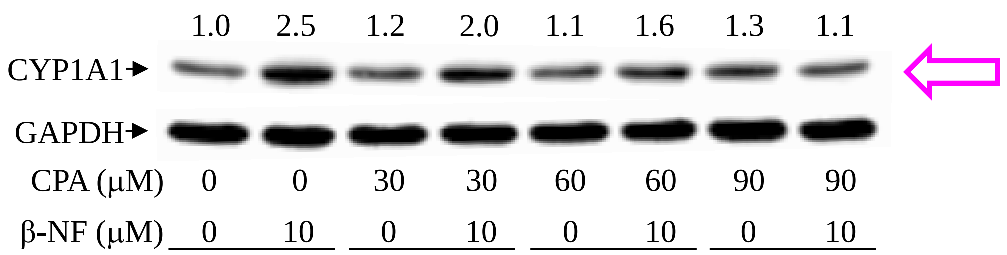

In order to decrease the volume of antibody, the blot was cut and the area of blot only corresponding to CYP1A1 or GAPDH was selected for hybridization individually.

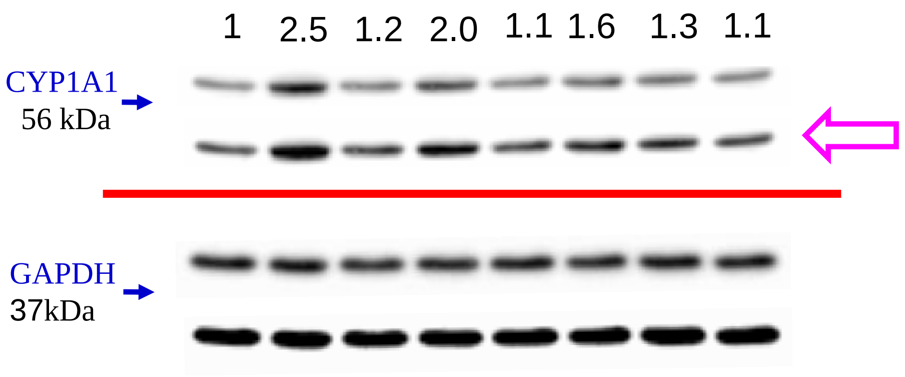

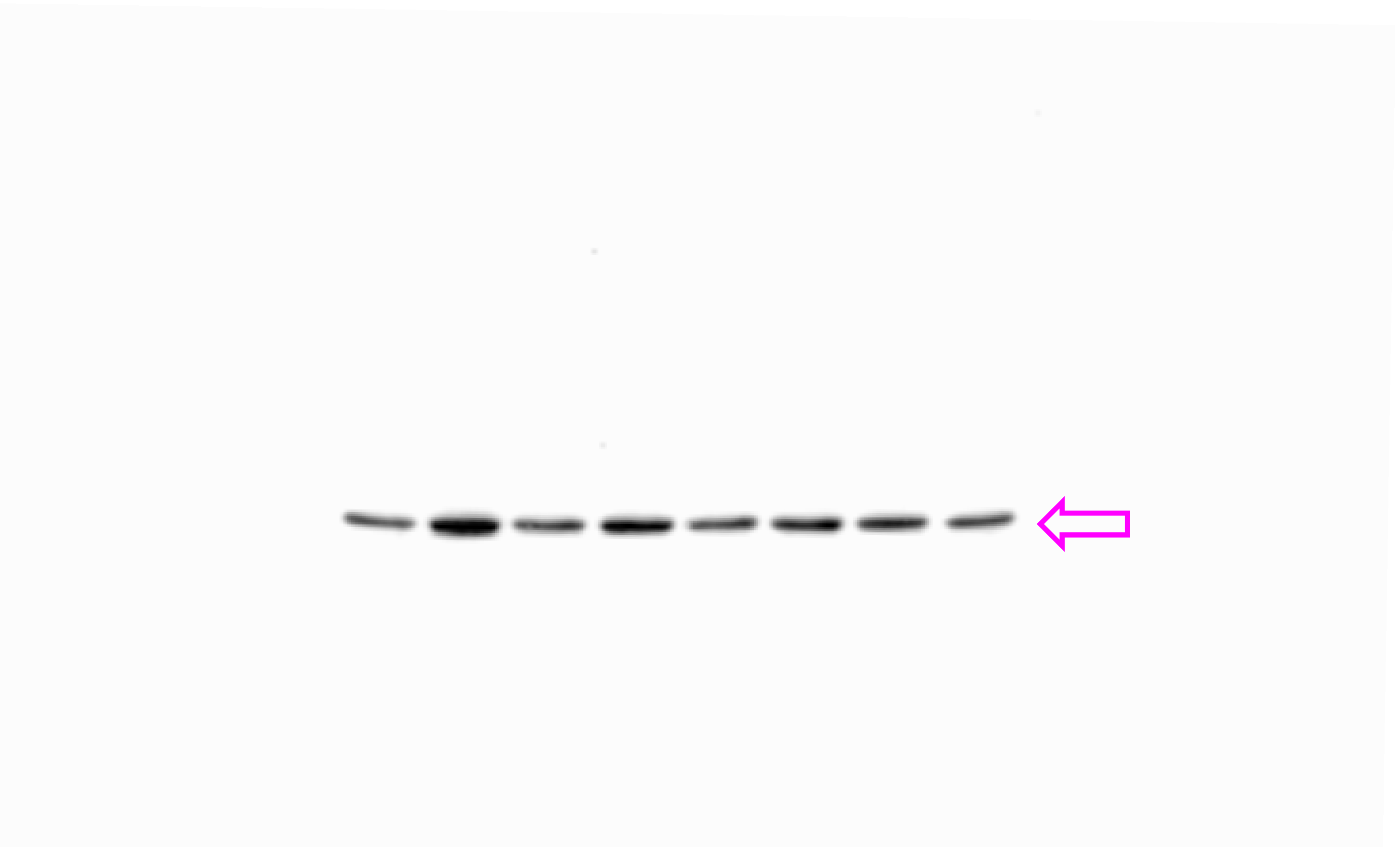

Fig. 7h (continued).

GAPDH h

p1

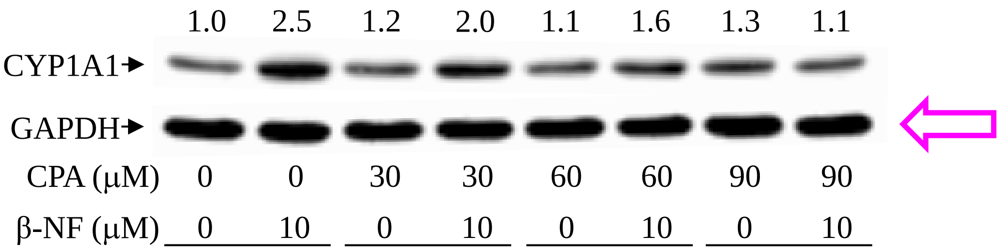

In order to decrease the volume of antibody, the blot was cut and the area of blot only corresponding to CYP1A1 or GAPDH was selected for hybridization individually.

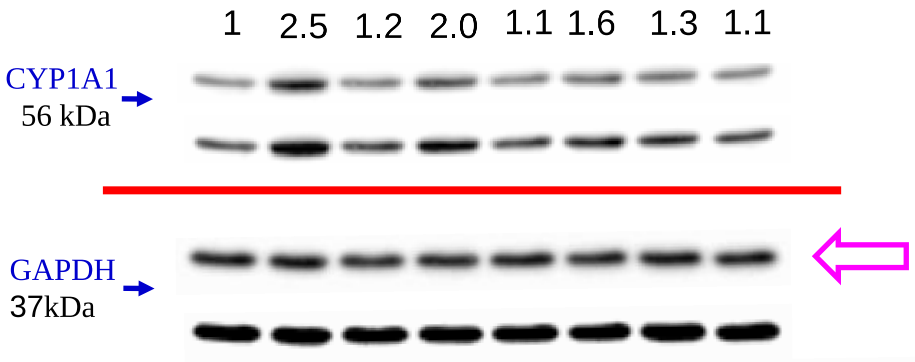

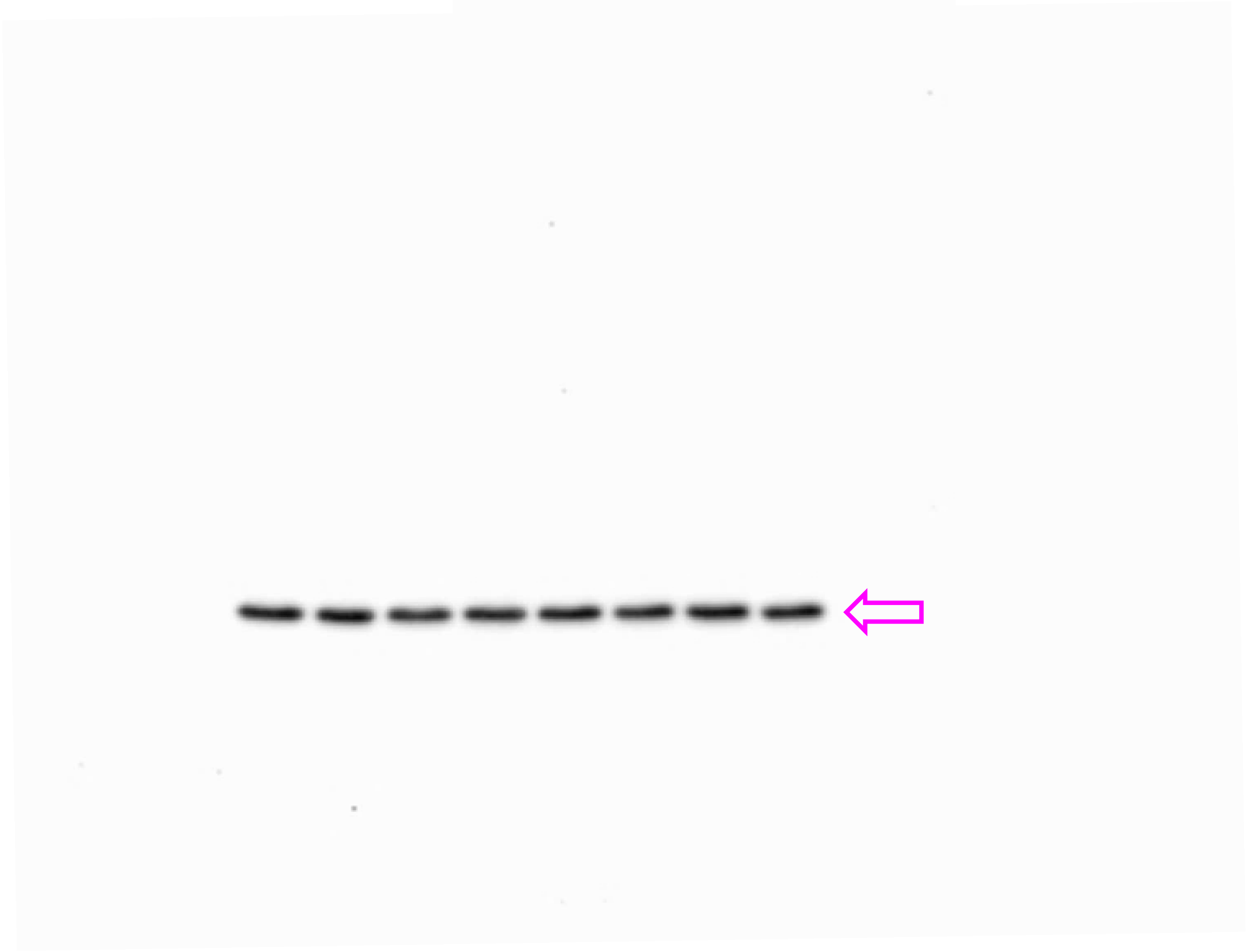

Fig. 7h (continued).

GAPDH

p2

h

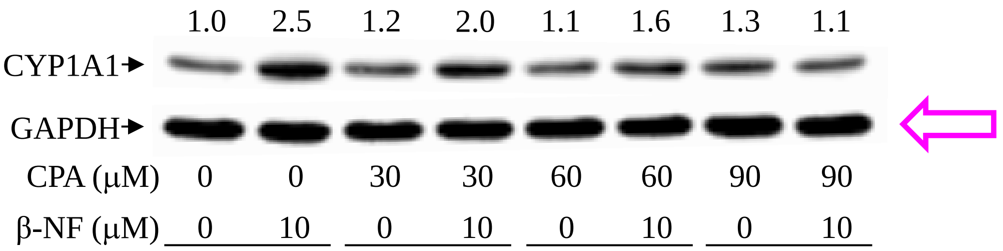

In order to decrease the volume of antibody, the blot was cut and the area of blot only corresponding to CYP1A1 or GAPDH was selected for hybridization individually.

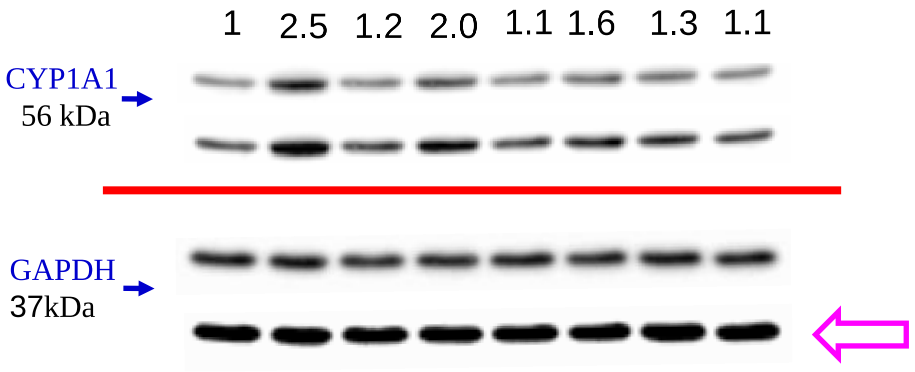

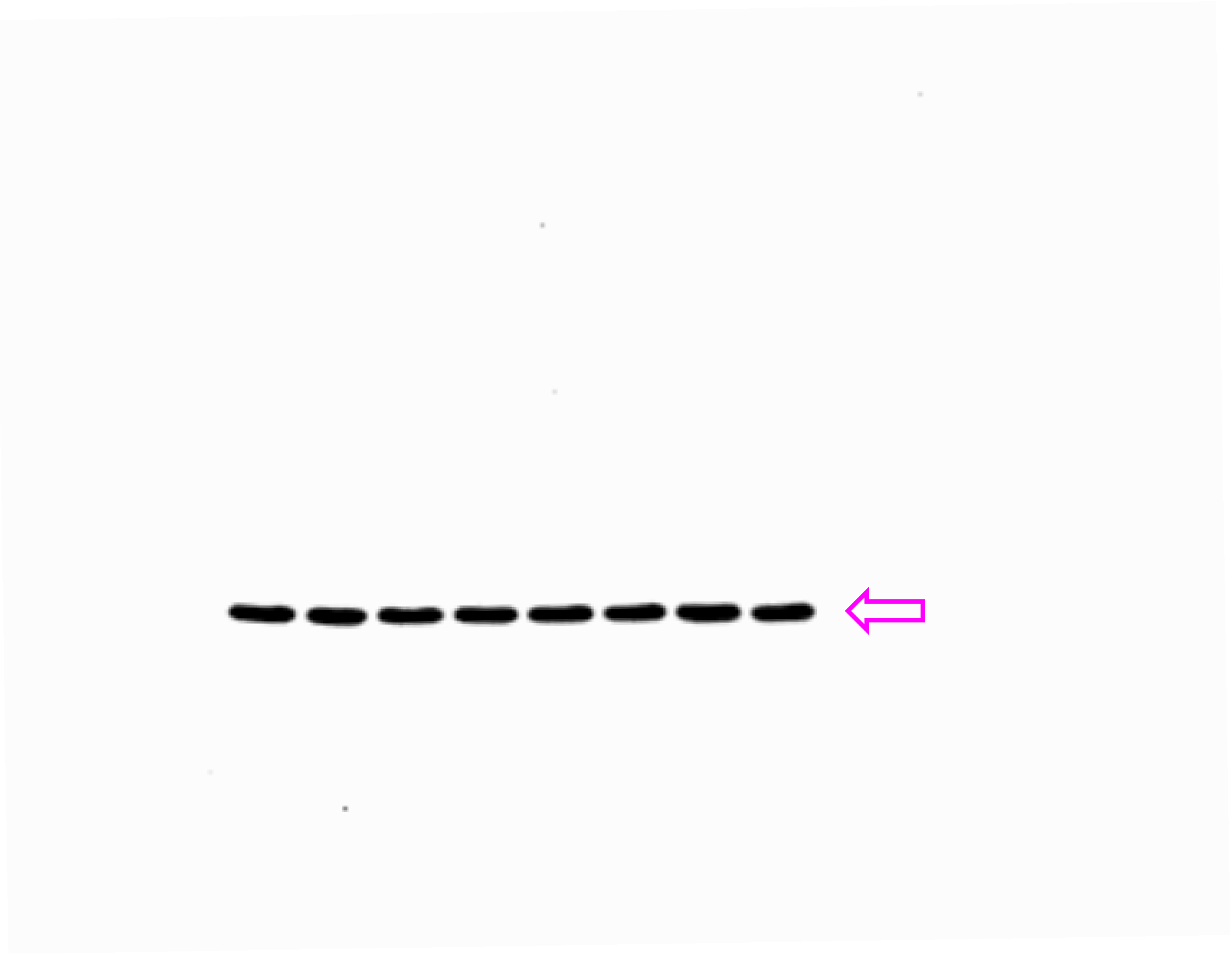

Fig. 7h (continued).

The Western blot for the same experiment in Fig. 7h was repeated here with membrane edges visible.

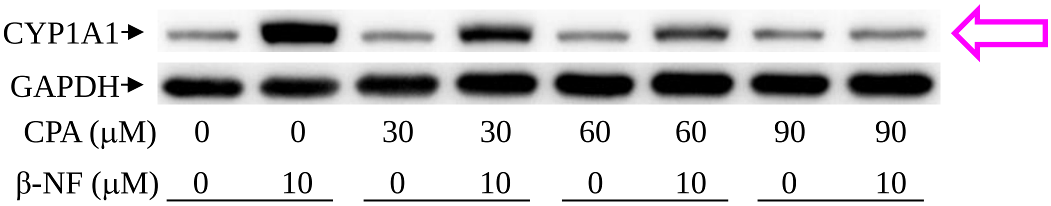

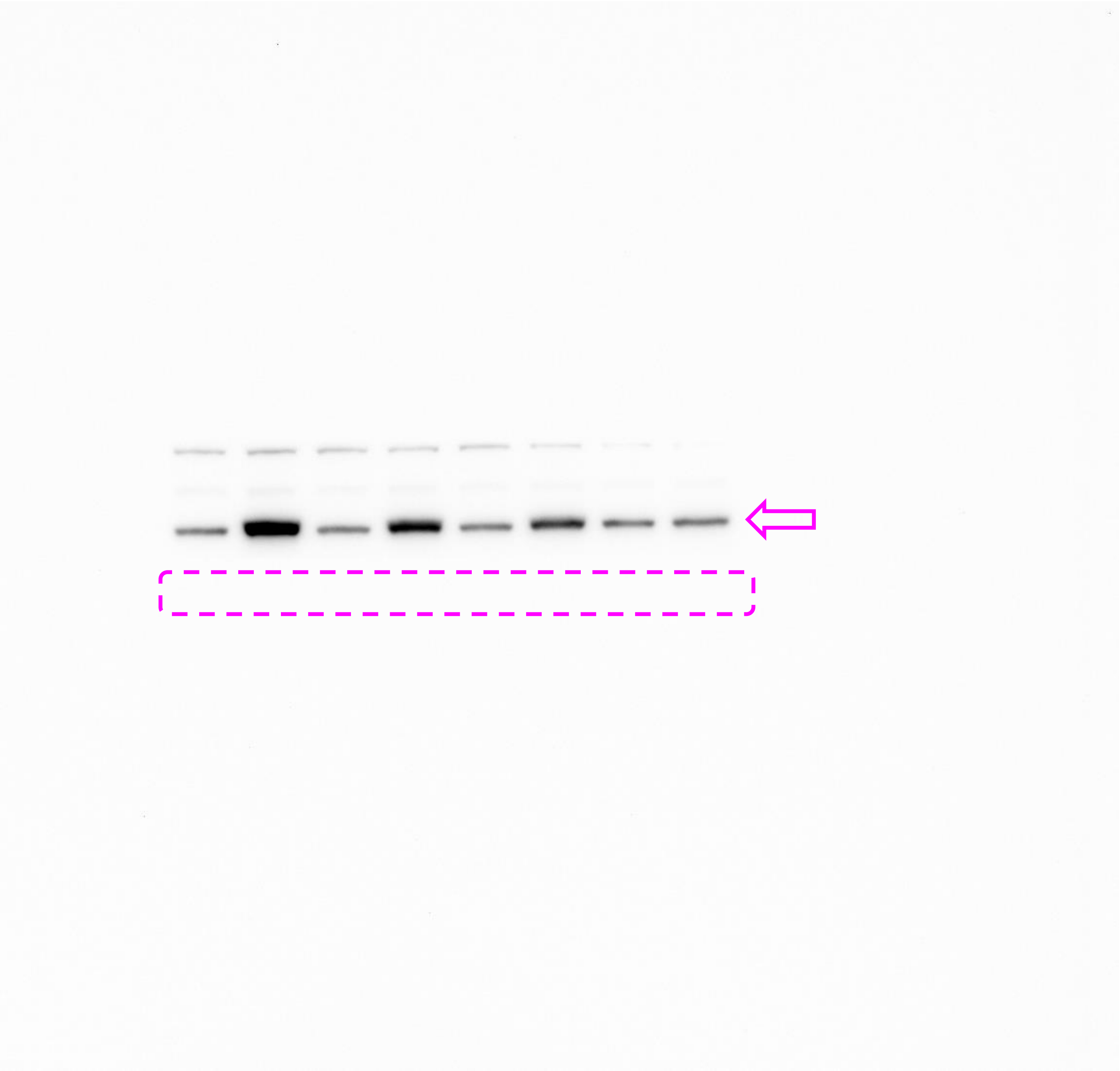

Fig. 7h (continued).
The Western blot for the same experiment in Fig. 7h was repeated here with membrane edges visible.

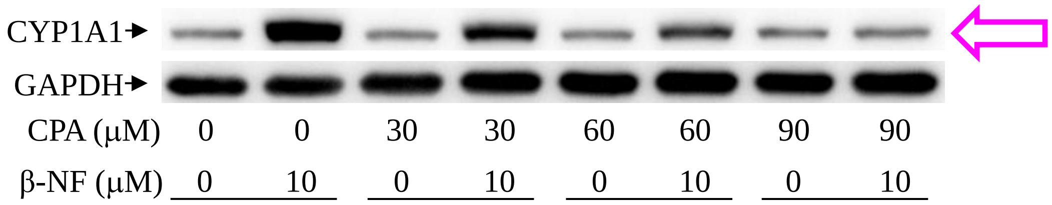

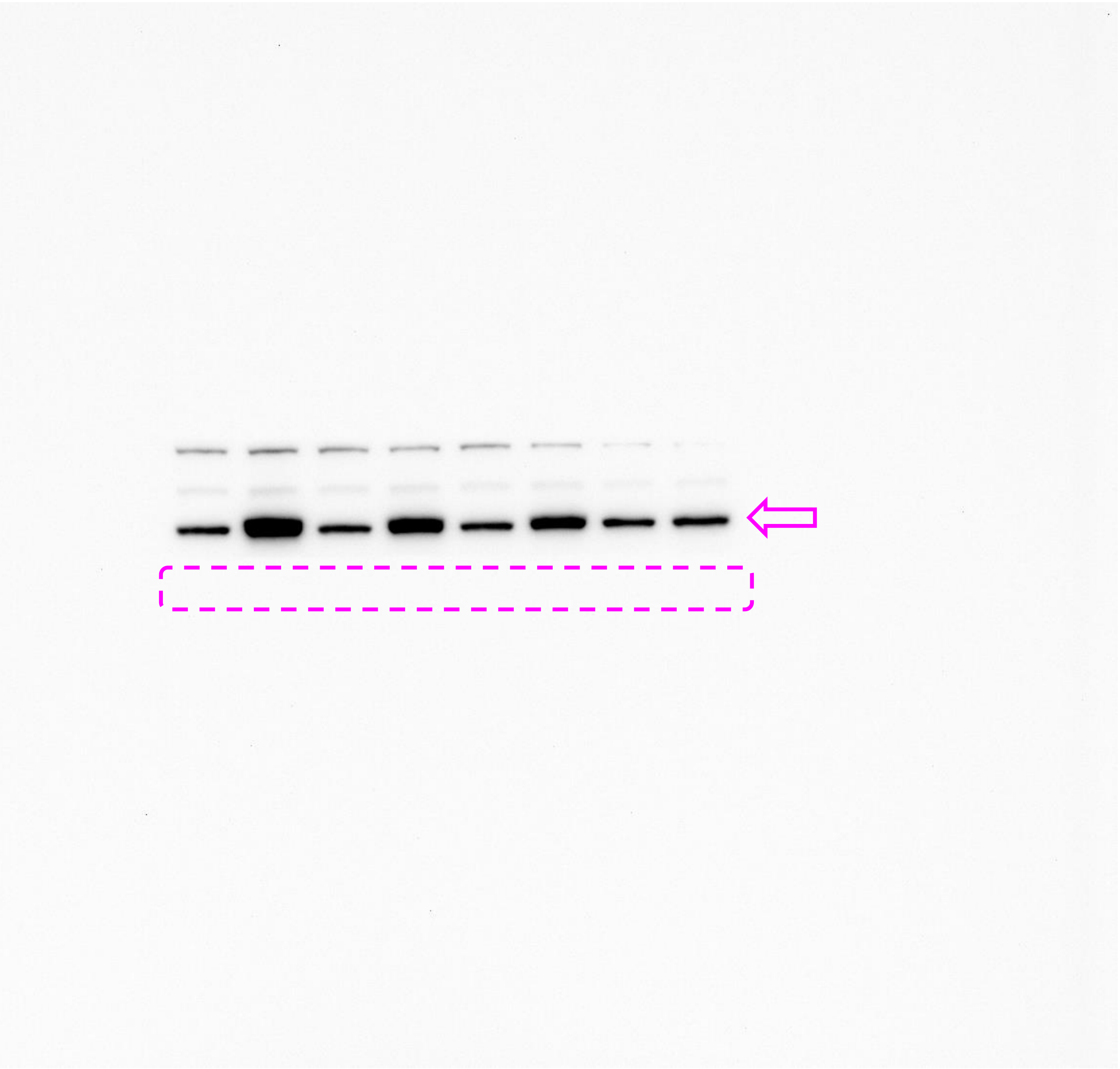

Fig. 7h (continued).

The Western blot for the same experiment in Fig. 7h was repeated here with membrane edges visible.

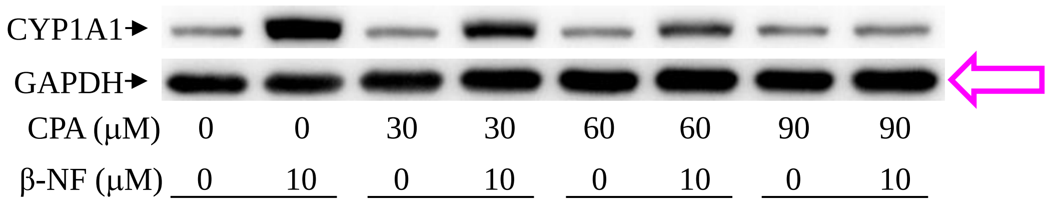

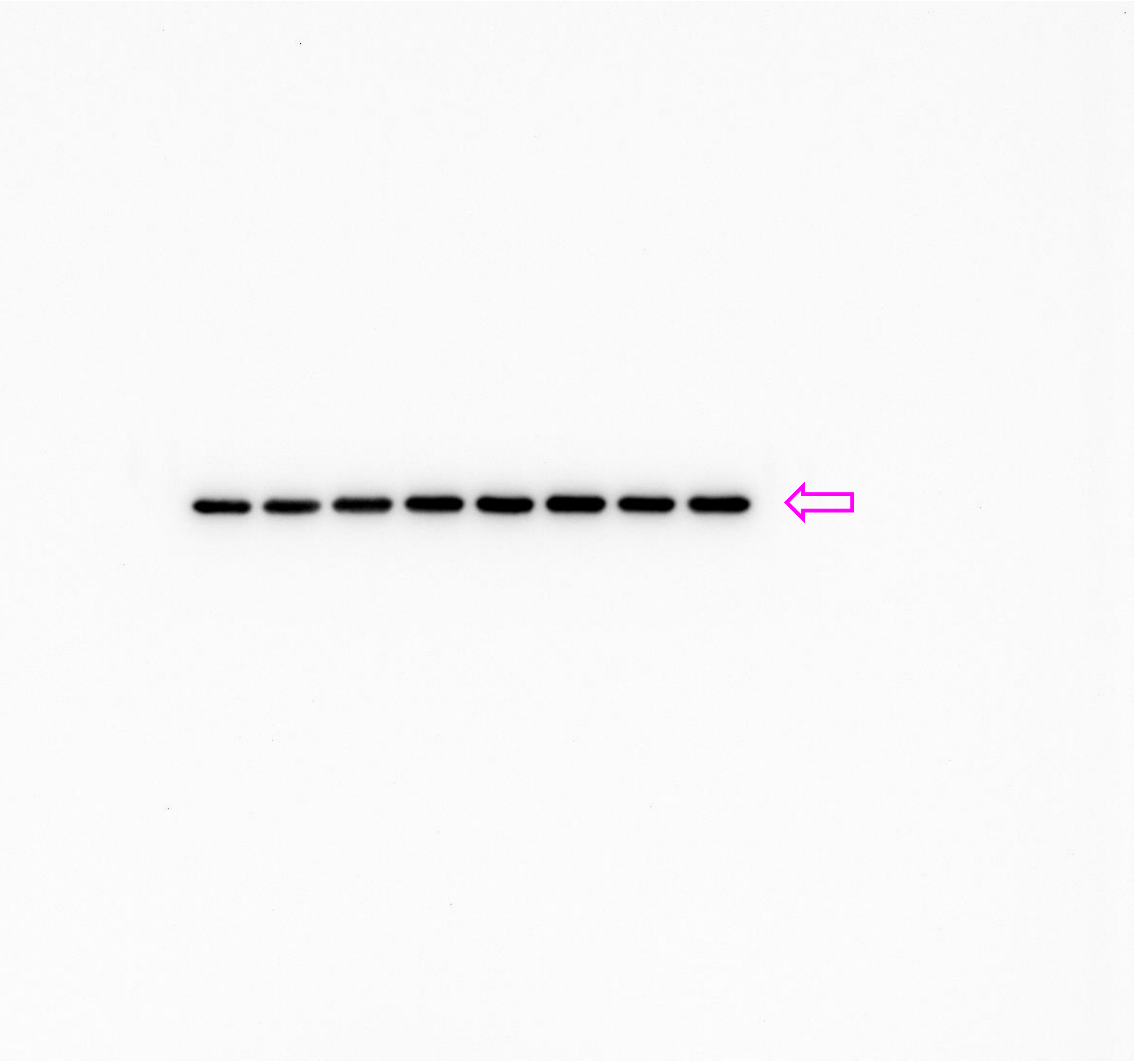

Fig. 7h (continued).

The Western blot for the same experiment in Fig. 7h was repeated here with membrane edges visible.

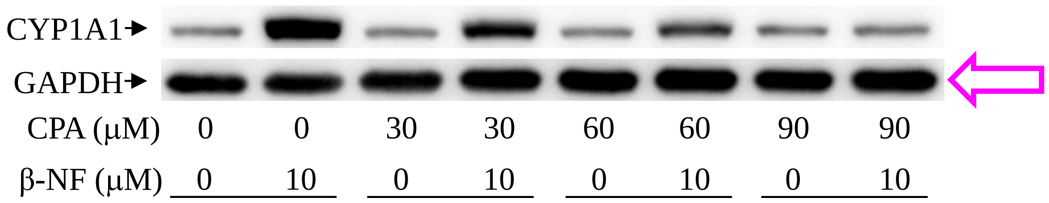

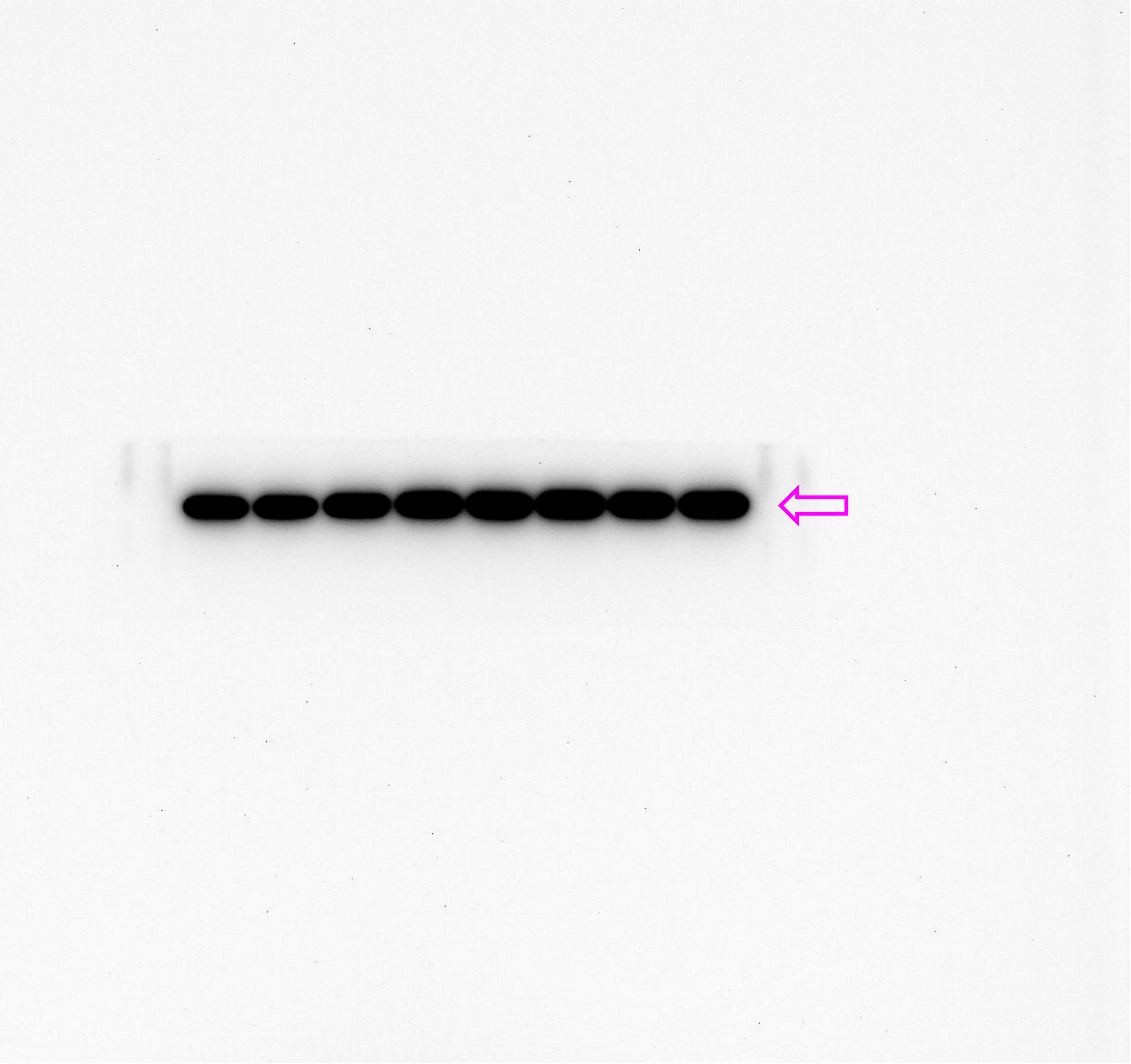

Supplement: Supplementary file 1 — Supplementary Information [file 41598_2021_84769_MOESM1_ESM.pdf]
